# Optimal Process Synthesis of Pesticide Production Considering Variable Demands and Raw Material Prices

**DOI:** 10.1021/acs.iecr.5c02394

**Published:** 2025-10-13

**Authors:** Austin Johnes, Faisal Khan, M. M. Faruque Hasan

**Affiliations:** †Artie McFerrin Department of Chemical Engineering, ‡Mary Kay O’Connor Process Safety Center, and §Texas A&M Energy Institute, 14736 Texas A&M University College Station, Texas 77843-3122, United States

## Abstract

In pesticide manufacturing, the handling and storage of hazardous chemicals are routinely performed under extreme conditions. Demand variability, particularly short-term and seasonal shifts, can exacerbate safety risks by increasing the need for extended storage of dangerous intermediates. Temporal and seasonal fluctuations in product demands as well as dynamic market prices of raw materials can greatly affect the cost-effectiveness, operational efficiency, and safety of pesticide production. To address these challenges, we present a superstructure flowsheet optimization approach for the selection of optimal processing routes to produce glyphosate—the world’s most widely used herbicide—under varying feedstock costs and product demand profiles. A hierarchical, bilevel superstructure representation allows for adjusting the fidelity of design while enabling simultaneous design and planning of pesticide production processes. We apply the approach for designing a glyphosate plant for meeting time-varying demands in the San Joaquin Valley of California. The results show that varying demand profiles significantly influence process design, affecting both the selection of chemical routes and storage requirements. Additionally, dynamic feed pricing introduces complex trade-offs, highlighting the need for an integrated approach toward future expansion to include both safety and economic considerations.

## Graphical abstract

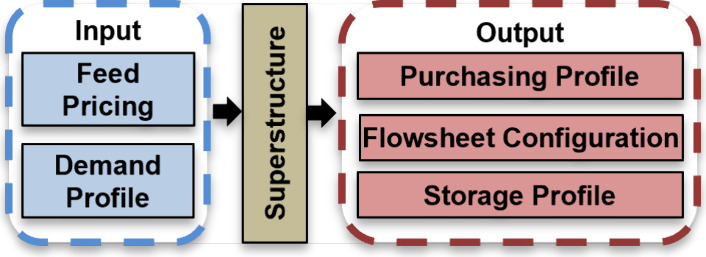

## 1 Introduction

Pesticides are classified as a broad range of chemicals typically designed to limit or remove unwanted pests in agriculture. Pesticides can range from insecticides that deal with insects, rodenticides that deal with rodents, and herbicides that deal with weeds. With a continual increase in the gross production value of agricultural products, it is expected that the market size for the pesticide industry will continue to grow.^1^ N-(phosphonomethyl)­glycine (PMG), more commonly known as glyphosate, is a pesticide primarily used as a herbicide with usages in many applications, ranging from homeowners to large-scale agricultural processes. In fact, glyphosate is also the most used herbicide in the world. As recently as 2014, over 800 million kilograms of glyphosate were applied annually worldwide.^2^

The production of pesticides is subject to variability in demand. For example, Figure 1a shows data from the California Department of Pesticide Regulation (CDPR) on pesticide applications in the year of 2022 for Fresno County.^3^ As the summer approaches, weekly pesticide demand increases due to increased use in agriculture. As the demand changes almost on a weekly basis, a glyphosate plant might suffer economic loss due to overproduction or not being able to satisfy customer demands due to underproduction. Variability in the purchase cost of the raw materials, as shown in Figure 1b, can also impact the overall economics of glyphosate production. As the demands and prices vary geographically, a process design that is optimal in one location may be economically infeasible at another. Therefore, it is important to design the pesticide production process keeping these in consideration.

**Figure 1:**
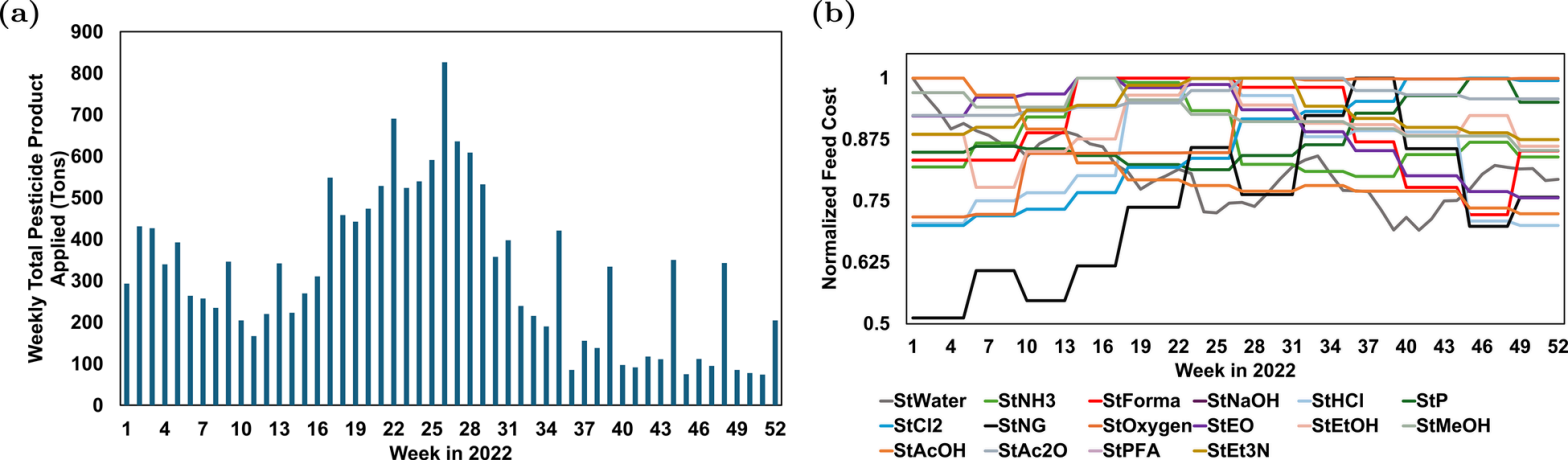
Representative data indicating weekly variation in pesticide demand and raw material prices: (a) weekly pesticide use in Fresno County, CA, in 2022; (b) normalized costs of various feedstocks for pesticide production in 2022.

In a pesticide process, many hazardous chemicals are processed and stored, often at extreme conditions, to obtain the desired product. Variable demands due to short-term and seasonal variations pose significant safety concerns due to the need for storing hazardous chemicals. As a result, process safety in the pesticide industry is of great importance. If safety is not ensured, serious accidents can happen. One such accident was in Bhopal, India, in 1984, where stored methyl isocyanate (MIC), a reactive intermediate in producing an insecticide, reacted with trace water in storage vessels, leading to a runaway exothermic reaction and the release of toxic chemicals into the atmosphere.^4,5^ As a result, thousands of people suffered injuries, and many were killed. A sister facility in Institute, West Virginia, was shut down due to concerns with the release of MIC after an explosion almost ruptured the tank storing MIC.^6^ Other incidents have occurred at pesticide facilities across the world, leading to injuries and death to many.^7,8^ Utilizing storage to mitigate costs and demand variability might introduce additional hazards. On the other hand, these variabilities could produce economically infeasible designs if only considering safety. Thus, before the question of how safety impacts decision-making can be addressed,^9^ a framework that integrates design and planning needs to be developed.

For integrated design and planning, instead of sequentially designing the process and then developing a schedule around the design, both are determined simultaneously in an optimization problem. A significant amount of research has been put forward examining the design and scheduling of processes as these are inherently time variant.^10–18^ Lin and Floudas^19^ utilized a state-task network to incorporate design, synthesis, and scheduling in the design of chemical plants. Beyond batch processes, work has been done examining systems with varying capacities based on availability of resources, such as electrolyzers and desalination systems, for simultaneous design and planning.^20,21^

There has been limited work focused on integrated design and planning of pesticide production processes. To capture the impact of variability in the design of pesticide processes, a holistic view of the system rather than individual operations is necessary. This is challenging as most work on pesticide processes has largely been centered around specific reactions and separation processes.^22–24^ To address these challenges, we propose a superstructure-based approach for design and planning under varying feed costs and product demand profiles. A superstructure-based process synthesis approach allows for the design of complex chemical processes using simpler unit blocks.^25^ One of the key advantages is the ability for decision-making while considering various complex interactions between processes.^26^ A superstructure represents all potential pathways that are plausible to produce a certain product. There is a wide variety of approaches in representing superstructure with varying levels of detail, capturing the underlying physical and chemical phenomena. The most well-known representations include the state-task network,^27^ the P-graph,^28^ the generalized modular framework,^29^ phenomena based approach,^30^ surrogate based approach,^31^ and a building block based approach.^32–34^

In this work, the overall superstructure model is represented using two tiers as levels, shown in Figure 2. Level 1 (L1) represents the major processing routes using key processing blocks and storage units. Level 2 (L2) considers the detailed unit operations within each block and accounts for the physical and chemical phenomena, such as reactors, separators, mixers, and movers. By separating the two levels, we can adjust the fidelity of process synthesis. This is critical as the level of fidelity of well-established versus emerging technologies can drastically differ in the design stage. By separating the decisions from the process flowsheets, we can integrate various levels of fidelity of models in a generalizable manner into the superstructure to see if novel routes can be found. The addition of storage to the L1 structure allows us to capture the impact of variability in the design decisions. The major contributions of this work are the following:

- a hierarchical, bilevel superstructure approach for process synthesis to adjust the fidelity of design accordingly based on the amount of readily available data,
- process design and optimization of industrial routes utilized for glyphosate production, and
- a framework for process synthesis under varying feed pricing and product demands.

**Figure 2:**
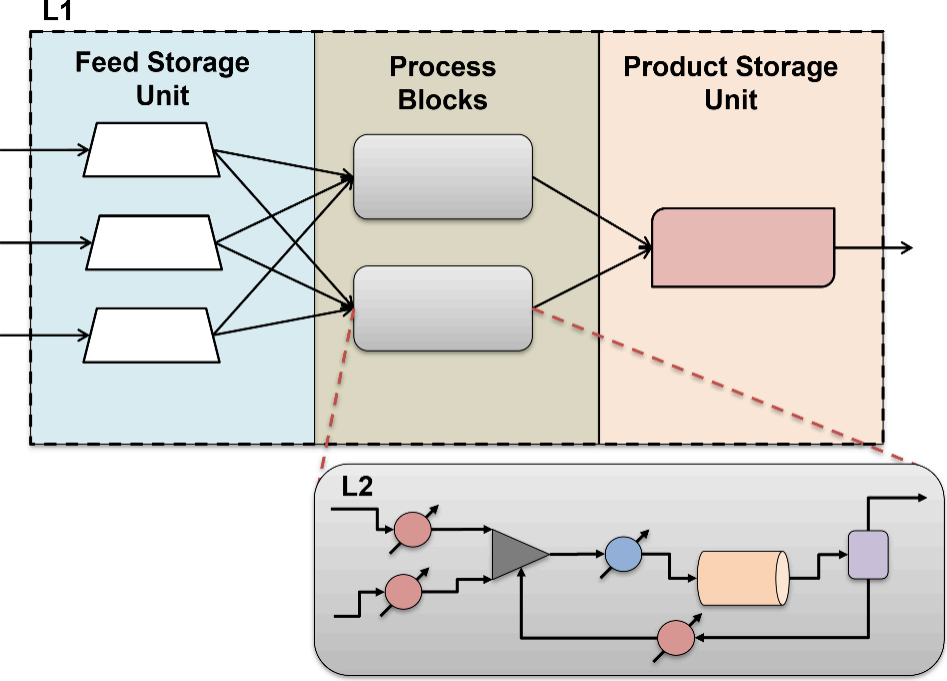
Overall two-tier superstructure approach.

The rest of the article is organized as follows. Section 2 describes the major processing routes for glyphosate production. Based on these routes, we also present a superstructure flowsheet that embeds all plausible process configurations for glyphosate production from various feedstocks. This also allows us to formulate a process synthesis model based on superstructure optimization in Section 3. A key feature of the proposed superstructure is that it allows higher-level (L1) design decisions considering big-picture goals and seasonal variations in feedstocks and product demands. At the unit operational level (L2), detailed fidelity-based process flowsheets are determined based on the L1 decisions. We apply this two-tier or bilevel process synthesis approach for designing a plant for meeting the time-varying glyphosate demand in the San Joaquin Valley of California based on representative data. Two different scenarios are considered and analyzed. The first is under the assumption that glyphosate production exactly matches the total product demand for every time period, where the demand is equivalent to the total product applied in the San Joaquin Valley for glyphosate products. The second scenario considers that the demand is equivalent to the total active ingredient applied in the San Joaquin Valley for glyphosate products. The active ingredient can be sold as both salts and solutions with varying concentrations. Final remarks and conclusions are presented in Section 5.

## 2 Glyphosate Pesticide Production Routes

Glyphosate can be synthesized in a variety of methods, but in industry, there are primarily three production routes: the hydrogen cyanide (HCN), diethanol­amine (DEA), and glycine routes.^35–37^ The HCN route was the first utilized to produce glyphosate at an industrial scale. Natural gas (NG), ammonia (NH_3_), and air are utilized to produce HCN, followed by a series of intermediates such as iminodiacetonitrile (IDAN), disodium iminodiacetate (DSIDA), phosphorus trichloride (PCl_3_), and N-(phosphonomethyl)­iminodiacetic acid (PMIDA). Through the conversion of the intermediates, glyphosate is eventually synthesized. The DEA route was developed later by Monsanto in order to reduce the amount of toxic chemicals used to produce DSIDA, namely the use of ammonia, hydrogen cyanide, and formaldehyde.^38–40^ Intermediates in the DEA route include DSIDA, PMIDA, and PCl_3_. The glycine route is the most recent of the production routes predominantly used in China.^40^ This route can take place at atmospheric pressure if desired. Unlike the HCN and DEA Routes, the glycine route does not have a series of intermediates that must be formed to produce glyphosate. To produce glyphosate via the glycine route, glycine and either dimethyl or diethyl phosphite (DMPP or DEPP) need to be synthesized or purchased.^40^

### 2.1 Hydrogen Cyanide (HCN) Route

The major steps necessary to produce glyphosate in the HCN route are shown in Figure 3a. More detailed processing configurations for all major steps are shown in Figure 4. Natural gas is mixed with air and ammonia. The mixture is then brought to over 1000 °C for the reaction to produce HCN.^41^ To remove the unreacted natural gas feedstock along with excess nitrogen and oxygen, an absorption tower separates the HCN into a liquid stream using water as the solvent (see Figure 4a). The excess water is then separated from the HCN by using a distillation tower with a partial condenser due to the presence of multiple noncondensables.

**Figure 3:**
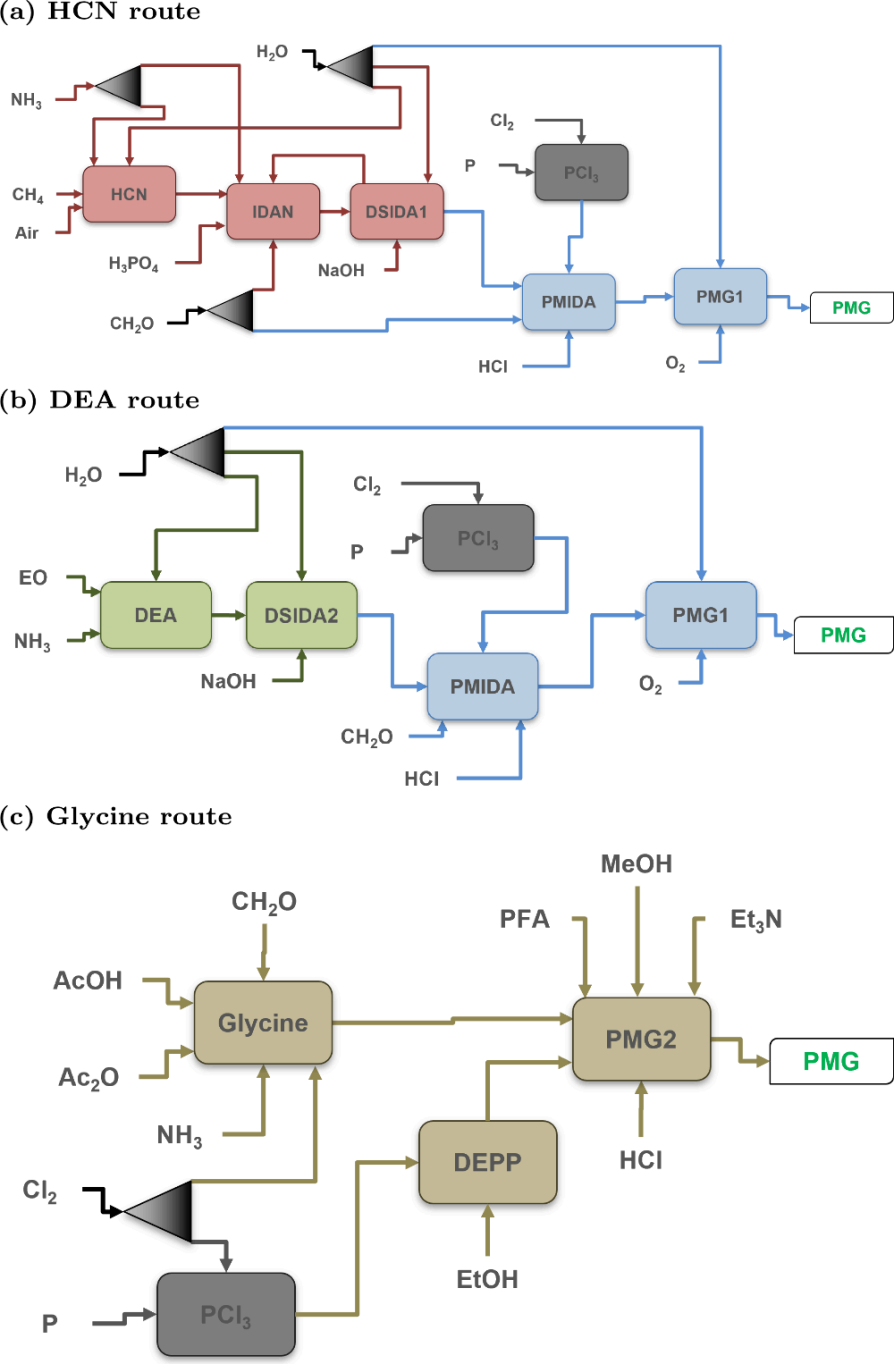
Block diagrams of major routes for glyphosate production: (a) HCN route, (b) DEA route, and (c) glycine route. These block diagrams show the level 1 (L1) structure of the glyphosate process.

**Figure 4:**
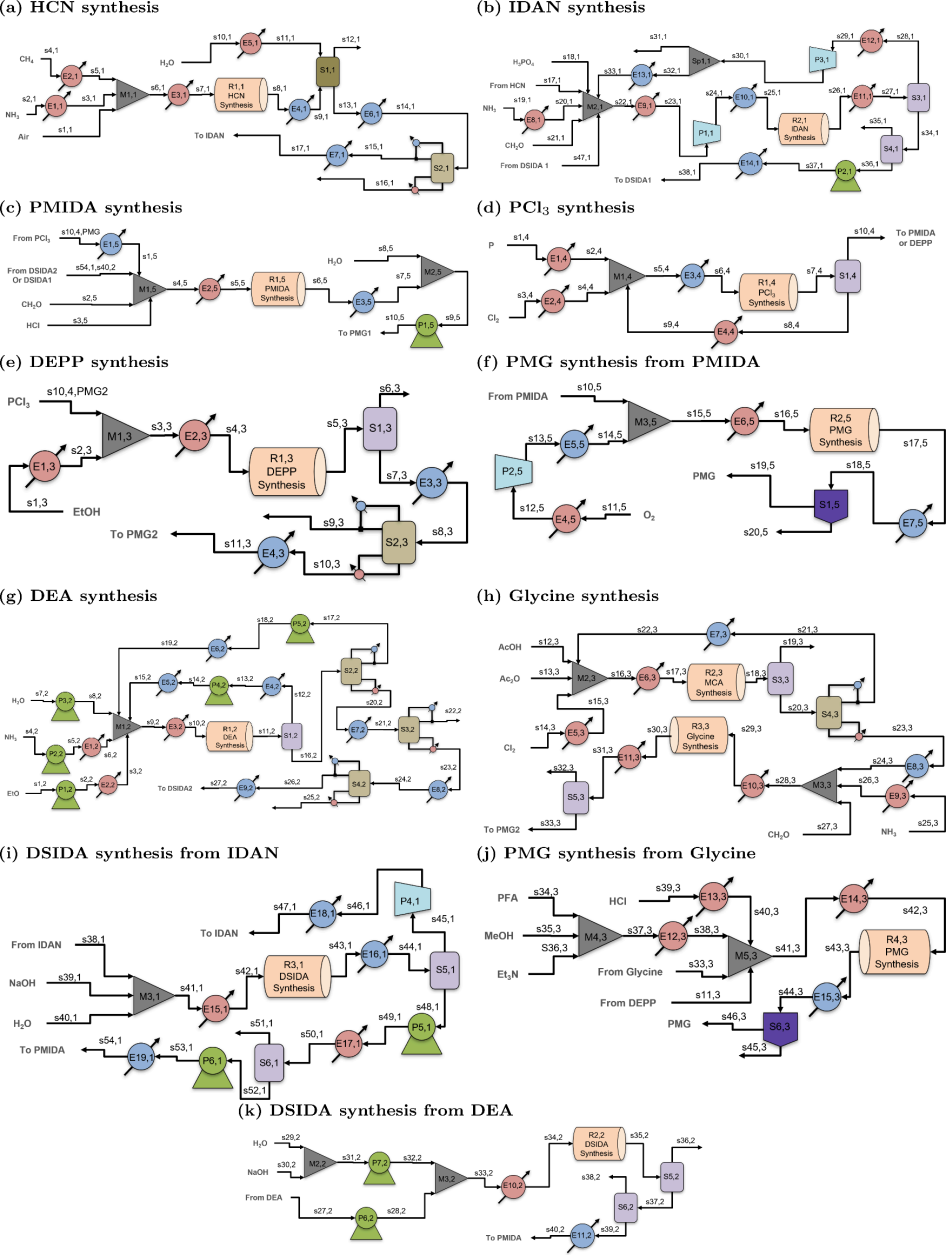
Process flowsheets for the major synthesis steps in various routes for glyphosate production: (a) HCN synthesis; (b) IDAN synthesis; (c) PMIDA synthesis; (d) PCl_3_ synthesis; (e) DEPP synthesis; (f) PMG synthesis from PMIDA; (g) DEA synthesis; (h) Glycine synthesis; (i) DSIDA synthesis from IDAN; (j) PMG synthesis from glycine; and (k) DSIDA synthesis from DEA. These flowsheets represent the level 2 (L2) structure of the glyphosate process.

HCN is then converted to IDAN, the first primary intermediate in the HCN route (Figure 4b). This is done by reacting HCN with formaldehyde (CH_2_O) and ammonia in the presence of an acid to catalyze the reaction at high pressure.^42^ In this reaction, unwanted byproducts, such as aminoacetonitrile, may be produced. To recycle some of the unused reactants, the product of the reactor is passed through a vapor–liquid equilibrium (VLE) separation unit, where a fraction of the vapor is sent back to the feed. The liquid is then sent through a flash tank to remove unwanted byproducts and chemicals present in the system before being sent to the DSIDA process. Unlike the flash vessels, the inlet stream of the VLE separators is already at a vapor–liquid equilibrium. For these vessels, no pressure drop is considered, and it is assumed that there is ample time for gravity-based separation of the vapor and liquid phases.

IDAN is then converted to a salt form, DSIDA, using sodium hydroxide (NaOH) (Figure 4i).^43^ One of the byproducts, formaldehyde, is separated in a flash tank under vacuum from the reactant mixture and sent to the IDAN process to help supplement the formaldehyde needed. The liquid from the flash is then sent to another flash tank under vacuum to remove any unwanted byproducts and chemicals present before being sent to the PMIDA process.

PCl_3_ is produced from the reaction between white and/or red phosphorus (P) and chlorine gas (Cl_2_), shown in Figure 4d.^44,45^ Unreacted reactants, along with some phosphorus trichloride, are recycled through the liquid bottom of a VLE separator, while the vapor top can be sent to either the PMIDA process from the DEA and HCN routes or the DEPP process from the Glycine route.

The products from the PCl_3_ and either of the two DSIDA processes are reacted along with formaldehyde in the presence of hydrochloric acid to produce PMIDA, the final intermediate in the HCN route (Figure 4c).^46^ Water is added to the product mixture to ensure the PMIDA is diluted in a solution before being fed to the PMG process.

At an elevated pressure, the PMIDA in the aqueous solution is reacted with pure oxygen (Figure 4f). Oxygen is initially fed into the system as liquid, where it is then heated to room temperature, compressed, and cooled to the same temperature as the aqueous PMIDA solution.^47^ After the glyphosate is produced, the solution is cooled so that glyphosate begins to precipitate out of the solution. The solution then goes through a filtration process where pure glyphosate can be obtained.^47^

### 2.2 Diethanol­amine (DEA) Route

The major steps to produce glyphosate in the DEA route are highlighted in Figure 3b along with the detailed flow diagrams for each section shown in Figure 4. Instead of using natural gas as feedstock, ethylene oxide (EO) is reacted with ammonia to produce a mixture of ethanol­amines. For selective production of DEA in the ethanol­amine mixture, the reaction takes place at extremely high pressures^48^ and in a certain ratio of ethylene oxide, ammonia, and water (see the process flow diagram for DEA synthesis in Figure 4g). Immediately after the reactor, a flash column is utilized to remove ammonia to be recycled back as feed. Since monoethanol­amine (MEA) and triethanol­amine (TEA) are not desired, a series of distillation columns is utilized to gather DEA.^49,50^ The first distillation column primarily removes ammonia to be recycled as feed. The second distillation column separates MEA as the top product, while DEA and TEA are the bottom products. The third distillation column separates DEA as the top product and TEA as the bottom product. The DEA is then sent to a DSIDA process (Figure 4k) that is different from the one described in the HCN route.

To produce DSIDA in the DEA route, DEA is dehydrogenated into DSIDA using sodium hydroxide.^51^ From this method to produce DSIDA, formaldehyde and hydrogen cyanide are eliminated at this step, while ammonia usage is minimized. The reactor product stream is then sent to a VLE separator and a flash tank to remove unwanted byproducts such as hydrogen and any excess chemicals.

Once DSIDA has been produced, PCl_3_ is produced in the same manner as in the HCN route (Figure 4d). PMIDA and PMG synthesis then follow the same process flowsheets as in the HCN route with a different stream composition (Figures 4c and 4f).

### 2.3 Glycine Route

The processes necessary to produce glyphosate in the glycine route are shown in Figure 3c. Once PCl_3_ has been produced in the same manner as presented in the HCN and DEA routes, either DMPP or DEPP is produced based on whether methanol (MeOH) or ethanol (EtOH) is used as the other reagent. In this process, ethanol and phosphorus trichloride are used to produce DEPP^52^ (see Figure 4e). After synthesis of DEPP, the product stream is initially put through a VLE separator to remove some of the vapor byproducts and unwanted chemicals. To further purify the DEPP, the liquid bottom of the VLE separator is sent to a distillation column, where the concentrated DEPP is received from the bottom of the distillation tower.

Glycine is produced (see Figure 4h) by first synthesizing monochloroacetic acid (MCA) using acetic acid (AcOH) and chlorine with acetic anhydride (Ac_2_O) present to catalyze the reaction.^53^ The unwanted byproduct, dichloroacetic acid (DCA), is removed via a VLE separator. The unreacted reactants and acetic anhydride are recycled via the top product of a distillation tower. The monochloroacetic acid is then reacted with ammonia to produce glycine.^54^ The produced glycine is then processed through a VLE separator to remove some of the unwanted byproducts and chemicals before being sent to the glyphosate process.

The synthesized glycine and DEPP produced are then fed into the glyphosate reactor along with hydrochloric acid, paraformaldehyde (PFA), triethylamine (EtN_3_), and methanol^55^ to produce PMG (see Figure 4j). It is assumed that the solid paraformaldehyde depolymerizes into formaldehyde as it is mixed with triethylamine and methanol along with no temperature change after mixing. Once glyphosate is produced, the stream is cooled to precipitate glyphosate from the solution before being fed into a filtration unit to separate glyphosate from the stream.

### 2.4 Glyphosate Process Superstructure

We combine all the synthesis routes in a process superstructure to present all of the plausible pathways for glyphosate production in a process flowsheet (Figure 5). Each colored block in the superstructure represents an entire process flowsheet that is utilized to produce a necessary intermediate to produce glyphosate (details are shown in Figures 4a-k). Blocks specific to each route are denoted with colors: red for HCN, green for DEA, and brown for glycine. Blocks that are common for all three routes are denoted in gray, and blocks that are utilized in only two out of three routes are denoted in blue. Potential feed storage blocks are denoted with a trapezoid and gray text, while potential product storage is denoted with green text. Stream connection blocks are denoted with a light blue text for intermediates and purple for the recycle. Additional purge streams are denoted with a light red text.

**Figure 5:**
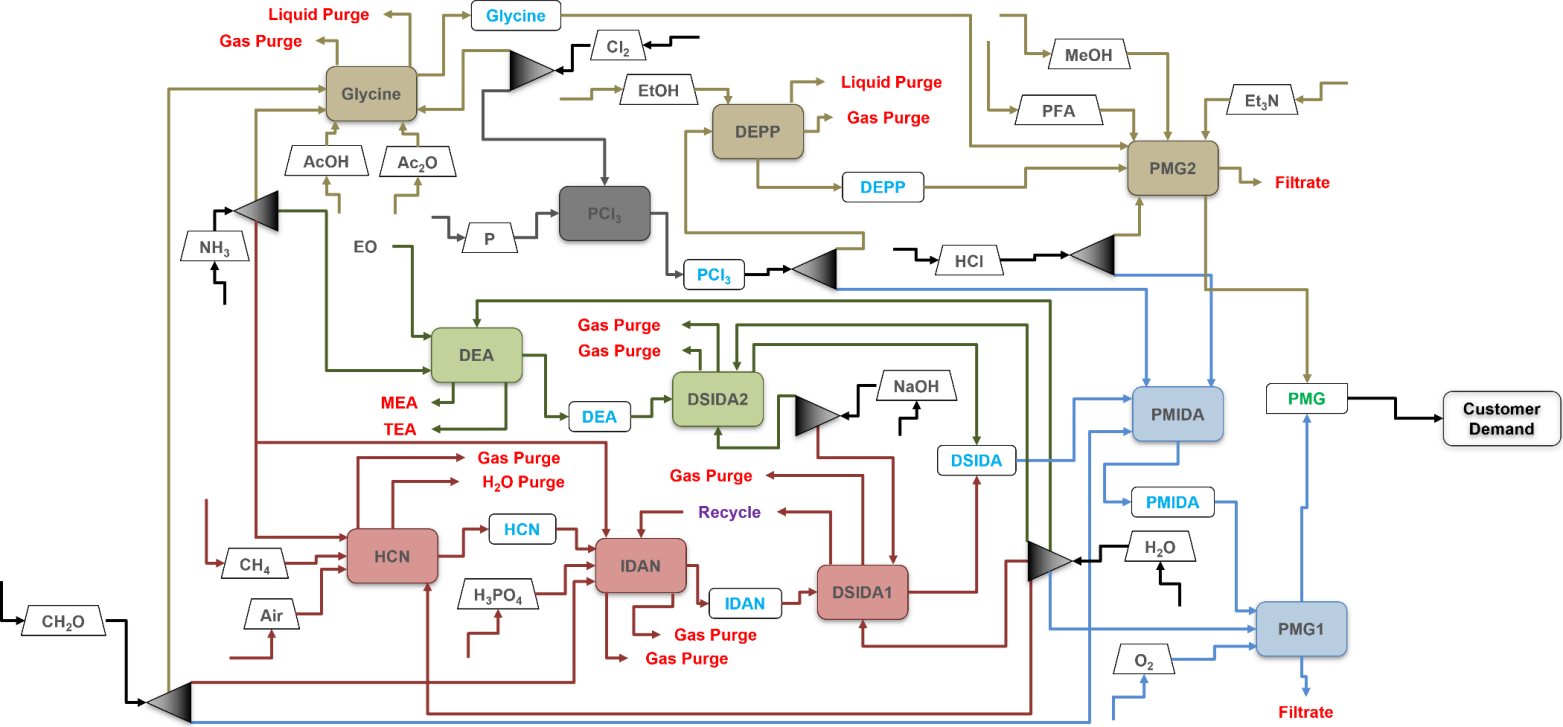
Glyphosate process superstructure showing all of the possible level 1 (L1) structural configurations. While the process blocks ensure that intermediate and final products are produced at a constant rate, the storage blocks ensure the variable demands are met over the entire year.

## 3 Model Formulation for Superstructure Optimization

Given the process superstructure in Figure 5, our goal is to synthesize the optimal processing route and determine the optimal chemical storage over time such that we are able to meet the time-varying demands of glyphosate in the most economical manner. To achieve this, we now present a mathematical model whose solution would determine the optimal route configuration for the production of glyphosate. Here, process streams are denoted with *i*, units/blocks with *j*, chemicals *c*, time-steps *t*, and technology routes τ. The model encompasses the flow of materials throughout the superstructure with the L1 and L2 structures. The L1 structure in Figure 3 consists of feed streams, primary process inlet, and outlet streams *i* ^L1^, blocks where unit operations take place *j* ^L1^, storage vessels, and chemicals *c*. The L1 structure is similar to a state-task network, where the streams represent the states and the blocks represent the tasks. In the L2 structure (Figure 4), inside each block streams *i* ^L2^ of chemicals *c* enter units *j* ^L2^. The L2 structure represents detailed physical and chemical phenomena in the process where operations such as separation, reaction, temperature change, and pressure change occur. Figure 6 shows the representation of the various types of flow connections and operations, both L1 and L2, which will be used for modeling purposes.

**Figure 6:**
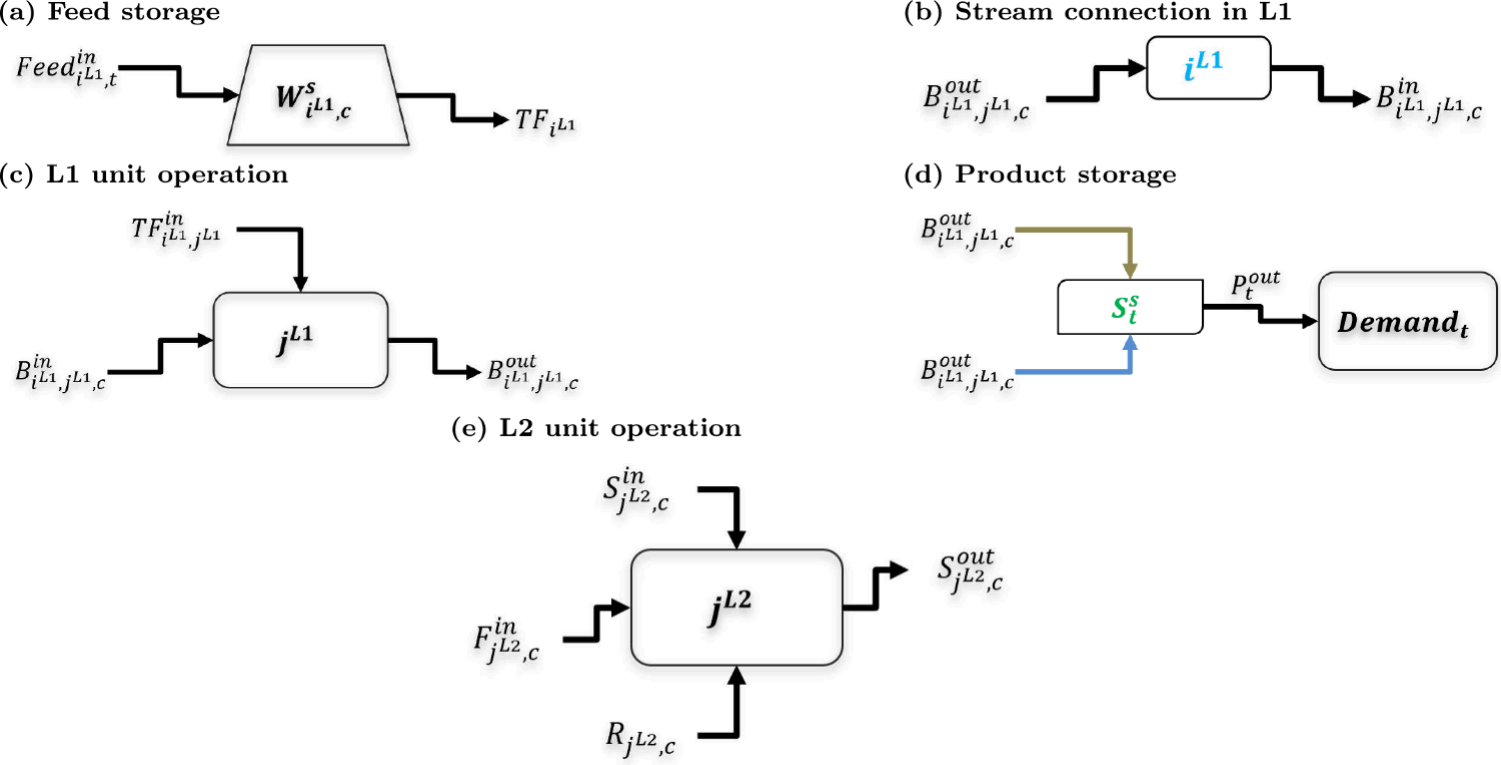
Representation of various blocks in the process superstructure: (a) feed storage; (b) L1 stream connection; (c) L1 unit operation; (d) product storage; and (e) L2 unit operation.

### 3.1 L1 Feed and Product Storage

Feed chemicals that enter the process plant in terms of a molar flow rate $Feed_{i^{L1},t}^{In}$ can be either immediately sent to be used in the continuous process $TF_{i^{L1}}$ or stored in $W_{i^{L1},t}^{s}$ to be used at a later time, as shown in Figure 6a. To address this, eqs 1 and 2 are utilized:

1 $$\left(Feed_{i^{L1},t-1}^{In}-TF_{i^{L1}}\right)\Delta T+W_{i^{L1},t-1}^{s}=W_{i^{L1},t}^{s},\forall i^{L1}\in I^{L1,Feed},\;t\in \left\{2,...,T\right\}$$

2 $$\left(Feed_{i^{L1},t}^{In}-TF_{i^{L1}}\right)\Delta T+W_{i^{L1},t}^{s}=W_{i^{L1}}^{inital},\forall i^{L1}\in I^{L1,Feed},\;t=\left|T\right|$$

where the amount stored in moles after the last time step in the period examined is denoted with $W_{i^{L1}}^{s,initial}$ . Since the composition does not change between purchasing, storage, and sending to the process, it does not need to be converted to a mass flow rate or indexed over the chemicals *c*. Additionally, Δ*T* is a parameter for the time-step in order to convert the molar flow rates to a total amount. To ensure that the amount stored initially is the same as the amount at the end, a cyclic constraint, eq 3, is added:

3 $$0=W_{i^{L1}}^{s,initial}-W_{i^{L1},t}^{s},\forall i^{L1}\in I^{L1,Feed},\;t=1$$

A desired product denoted by the subset *C* ^*product*^ exits only from specific L1 streams and units, denoted by the subset $J_{i^{L1}}^{L1,Product,Match}$ . It can be either sent directly to satisfy customer demands $P_{t}^{Out}$ in terms of a molar flow rate or stored for later use $S_{t}^{s}$ as shown in Figure 6d. The overall balance, eqs 4 and 5, is similar to the feed component storage balance:

4 $$\left(\underset{i^{L1}\in I^{L1}}{\sum }\underset{j^{L1}\in J_{i^{L1}}^{L1,Product,Match}}{\sum }\underset{c\in C^{Product}}{\sum }B_{i^{L1},j^{L1},c}^{Out}-P_{t-1}^{Out}\right)\Delta T+S_{t-1}^{s}=S_{t}^{s},\forall t\in \left\{2,...,T\right\}$$

5 $$\left(\underset{i^{L1}\in I^{L1}}{\sum }\underset{j^{L1}\in J_{i^{L1}}^{L1,Product,Match}}{\sum }\underset{c\in C^{Product}}{\sum }B_{i^{L1},j^{L1},c}^{Out}-P_{t}^{Out}\right)\Delta T+S_{t}^{s}=S^{s,initial},\forall t=\left|T\right|$$

Like with the feed storage, a cyclic, eq 6, is added to ensure the amount of product stored at the first time step is equal to the amount stored at the beginning of the next time period *S* ^*s*,*initial*^:

6 $$0=S^{initial}-S_{t}^{s},t=1$$

The amount that is sent to satisfy the customer’s demand must be equal to the customer’s molar demand *Demand*_*t*_, shown through eq 7:

7 $$Demand_{t}=P_{t}^{Out},\forall t\in T$$

### 3.2 Level 1 Decisions

#### 3.2.1 L1 Mole Balances

Entry of stream *i* ^L1^ into a block *j* ^L1^ can be from either a molar feed stream $TF_{i,j}^{in,L1}$ or a molar process inlet stream $B_{i^{l1},j^{L1}}^{In}$ , as shown in Figure 6c. The total molar feed stream $TF_{i^{L1}}$ is defined as the sum of blocks *j* ^L1^ that stream *i* ^L1^ enters. Therefore, we have in eq 8,

8 $$TF_{i^{L1}}=\underset{j^{L1}\in J_{i^{L1}}^{Feed,L1}}{\sum }TF_{i^{L1},j^{L1}}^{in,L1},\forall i^{L1}\in I^{L1,feed}$$

The subset $J_{i^{L1}}^{Feed,L1}$ denotes all L1 blocks to which a given feed stream in the L1 structure is connected. The subset *I* ^L1,*feed*^ constrains feed balance to only streams in the L1 structure that are feed streams. These inlets stream to block *j* ^L1^ of the superstructure connect to specific process streams within the L2 structure $F_{i^{L2}}$ as shown in eq 9:

9 $$TF_{i^{L1},j^{L1}}^{in,L1}=\underset{i^{L2}\in I_{i^{L1},j^{L1}}^{Feed,L1,L2}}{\sum }F_{i^{L2}},\forall i\in I^{L1},\;j\in J_{i^{L1}}^{Feed,L1}$$

The subset $I_{i^{L1},j^{L1}}^{Feed,L1,L2}$ denotes all feed inlet streams in the L2 structure that are connected to a match of stream and unit in the L1 structure. For process streams, the molar inlet of a process stream into a block $B_{i^{L1},j^{L1},c}^{In}$ is equal to the sum of all potential streams $S_{i^{L2},c}^{c}$ connected in L2. Therefore, in eq 10,

10 $$B_{i^{L1},j^{L1},c}^{In}=\underset{i^{L2}\in I_{i^{L1},j^{L1}}^{Process,In,L1,L2}}{\sum }S_{i^{L2},c}^{c},\forall i^{L1}\in I_{j^{L1}}^{Inlet,L1},\;j^{L1}\in J^{L1},\;c\in C$$

The subset $I_{i^{L1},j^{L1}}^{Process,In,L1,L2}$ denotes all process inlet streams in the L2 structure that are connected to a match of stream and unit in the L1 structure. The subset $I_{j^{L1}}^{Inlet,L1}$ constrains the main balance to only streams in the L1 structure that are process streams that can be connected to the unit in the L1 structure. Similarly, the molar outlet stream $B_{i^{L1},j^{L1},c}^{Out}$ of a L1 block is equal to the sum of all potential streams $S_{i^{L2},c}^{c}$ in L2 that is connected to stream *i* ^L1^ and block *j* ^L1^. This allows us to ensure the following in eq 11:

11 $$B_{i^{L1},j^{L1},c}^{Out}=\underset{i^{L2}\in I_{i^{L1},j^{L1}}^{Process,Out,L1,L2}}{\sum }S_{i^{L2},c}^{c},\forall i^{L1}\in I_{j^{L1}}^{Outlet,L1,Out},\;j^{L1}\in J^{L1},\;c\in C$$

The subset $I_{i^{L1},j^{L1}}^{Process,Out,L1,L2}$ denotes all process outlet streams in the L2 structure that are connected to a match of stream. The outlet process stream *i* ^L1^ for a block *j* ^L1^ might be the inlet process stream for multiple other blocks and vice versa, as shown in Figure 6b. Thus, it is defined that the sum of the outlet $B_{i^{L1},j^{L1},c}^{Out}$ is equal to the sum of all other blocks that it enters $B_{i^{L1},j^{L1},c}^{In}$ . With these, we can write eq 12:

12 $$\underset{j^{L1}\in J_{i^{L1}}^{Outlet,L1}}{\sum }B_{i^{L1},j^{L1},c}^{Out}=\underset{j^{L1}\in J_{i^{L1}}^{Inlet,L1}}{\sum }B_{i^{L1},j^{L1},c}^{In},\forall i\in I^{L1,Process},\;c\in C$$

The subsets $J_{i^{L1}}^{Outlet,L1}$ denote each unit that serves as an outlet unit for a certain process stream, while the subset $J_{i^{L1}}^{Inlet,L1}$ denotes each unit that serves as an inlet unit for a certain process stream.

#### 3.2.2 L1 Mass Balances

To ensure mass is conserved across both streams and the entire superstructure, eqs 13 and 14 are added:

13 $$\underset{c\in C}{\sum }\underset{j^{L1}\in J_{i^{L1}}^{Outlet,L1}}{\sum }MW_{c}\;B_{i^{L1},j^{L1},c}^{Out}=\underset{c\in C}{\sum }\underset{j^{L1}\in J_{i^{L1}}^{Inlet,L1}}{\sum }MW_{c}\;B_{i^{L1},j^{L1},c}^{In},\forall i\in I^{L1,Process}$$

14 $$\underset{c\in C}{\sum }\underset{i^{L1}\in I_{j^{L1}}^{Feed,L1}}{\sum }MW_{c}\;z_{i^{L1},c}^{L1}TF_{i^{L1},j^{L1}}^{in,L1}+\underset{c\in C}{\sum }\underset{i^{L1}\in I_{j^{L1}}^{Inlet,L1}}{\sum }MW_{c}\;B_{i^{L1},j^{L1},c}^{In}=\underset{c\in C}{\sum }\underset{i^{L1}\in I_{j^{L1}}^{Outlet,L1,Out}}{\sum }MW_{c}\;B_{i^{L1},j^{L1},c}^{Out},\forall j^{L1}\in J^{L1}$$

where *MW*_*c*_ is the molecular weight of each chemical *c* and $z_{i^{L1},c}^{L1}$ is the inlet feed compositions. Additionally, the subset $I_{j^{L1}}^{Feed,L1}$ is equivalent to $J_{i^{L1}}^{Feed,L1}$ as this is stream-unit matching.

### 3.3 Level 2 Decisions

#### 3.3.1 L2 Mass Balances

Based on varying amounts of data available, the L2 structure can be formulated accordingly. For example, if only the feed in and the product out of each block were known, the L2 structure could be treated as a simple in–out block. For the case where the units are known along with fixed temperatures and pressures, the following L2 model can be utilized.

First, conservation of mass must be ensured across each of the units *j* ^L2^. For each unit *j* ^L2^ shown in Figure 6e, the inlet molar flow can be converted to a mass flow with eq 15:

15 $$F_{j^{L2},c}^{In}=MW_{c}\underset{i^{L2}\in I_{j^{L2}}^{Feed,In,Set}}{\sum }z_{i^{L2},c}\;F_{i^{L2}},\forall j^{L2}\in J^{L2},\;c\in C$$

In a given unit, there might be multiple streams *i* ^L2^ either entering or leaving the unit while various chemicals *c* are entering, leaving, reacting, and/or produced. Streams that enter a unit can either come from other units or enter directly as feed material from storage. For feed entering from storage, the total feed is denoted as $F_{i^{L2}}^{L2}$ where $z_{i^{L2},c}$ is a parameter for the known composition of each chemical in the feed and *MW*_*c*_ is the molecular weight of each component. To ensure that only streams that enter that unit are in the summation, the subset $I_{j^{L2}}^{Feed,In,Set}$ identifies the feed streams for a unit of the L2 structure. The summation of each of these streams entering a unit for a chemical of technology is denoted by $F_{j^{L2},c}^{In}$ .

Let $S_{i^{L2},c}^{c}$ denote the molar flow rate of a chemical in a stream in the L2 structure. The subset $I_{j^{L2}}^{Stream,In,Set}$ ensures that only streams that should enter a unit are included in the summation. Using this and the molecular weight *MW*_*c*_, the total molar flow into a unit from streams coming from other units is converted to a mass flow $S_{j^{L2},c}^{In}$ . Therefore, we have the following in eq 16:

16 $$S_{j^{L2},c}^{In}=MW_{c}\underset{i^{L2}\in I_{j^{L2}}^{Stream,In,Set}}{\sum }S_{i^{L2},c}^{c},\forall j^{L2}\in J^{L2},\;c\in C$$

The total amount of mass of a chemical exiting a unit $S_{j^{L2},c}^{Out}$ can exit into multiple streams, such as the case of a flash tank. To address this, the subset $I_{j^{L2}}^{Stream,Out,Set}$ ensures that streams that are the outlet of a unit are included in the summation with the following in eq 17:

17 $$S_{j^{L2},c}^{Out}=MW_{c}\underset{i^{L2}\in I_{j^{L2}}^{Stream,Out,Set}}{\sum }S_{i^{L2},c}^{c},\forall j^{L2}\in J^{L2},\;c\in C$$

For units involving chemical reactions, it is approximated that each reaction that takes place is a stoichiometric yield-based reaction. The amount of limiting reagent that enters the unit is denoted by $LR_{j^{L2}}$ . Two subsets are necessary to determine the amount of limiting reagent. The first subset is $I_{j^{L2}}^{Stream,In,Set}$ as mentioned earlier and $C_{j^{L2}}^{LR,Set}$ , which ensures that the only chemical included in the summation is the limiting reagent. For the flowsheets developed, no feed directly enters a reaction unit, and thus the summation with the subset $I_{j^{L2}}^{Feed,In,Set}$ is not needed in the equation. For units where no reaction takes place, no stream is included in the summation, so the amount of limiting reagent is equal to zero. We ensure the conversion as shown in eq 18:

18 $$LR_{j^{L2}}=\underset{c\in C_{j^{L2}}^{LR,Set}}{\sum }\underset{i^{L2}\in I_{j^{L2}}^{Stream,In,Set}}{\sum }S_{i^{L2},c}^{c},\forall j^{L2}\in J^{L2}$$

Since it is assumed each reaction is a stoichiometric yield-based reaction, a conversion factor is utilized $\chi_{j^{L2},c}$ . Additionally, the sign of the conversion factor is negative if it is in reference to a reactant and positive if it is in reference to a product. Using the amount of limiting reagent entering a reaction unit, the total mass amount of chemical either reacted or produced for a technology is determined $R_{j^{L2},c}$ as shown in eq 19:

19 $$R_{j^{L2},c}=MW_{c}\;LR_{j^{L2}}\;\chi_{j^{L2},c},\forall j^{L2}\in J^{L2},\;c\in C$$

Using the above equations, the overall chemical mass balance for a unit can be determined. If there is a reaction taking place, then eq 20 is utilized:

20 $$F_{j^{L2},c}^{In}+S_{j^{L2},c}^{In}+R_{j^{L2},c}-S_{j^{L2},c}^{Out}=0,\forall j^{L2}\in J^{L2,Rxn},\;c\in C$$

where *J* ^L2,*Rxn*^ denotes the subset of L2 reaction units. For L2 units where no reaction takes place, the subset *J* ^L2,*No*,*Rxn*^ is utilized in eq 21:

21 $$F_{j^{L2},c}^{In}+S_{j^{L2},c}^{In}-S_{j^{L2},c}^{Out}=0,\forall j^{L2}\in J^{L2,No,Rxn},\;c\in C$$

In addition, to ensure total conversion of mass across a unit, the overall mass balance in eq 22 is also included to make sure the total mass that enters the unit is the same that exits:

22 $$0=\underset{c\in C}{\sum }F_{j^{L2},c}^{In}+\underset{c\in C}{\sum }S_{j^{L2},c}^{In}-\underset{c\in C}{\sum }S_{j^{L2},c}^{Out},\forall j^{L2}\in J^{L2}$$

#### 3.3.2 L2 Mole Balances

With the mass balance for each unit conserved from the previous set of equations, the equations for separation utilized are in terms of molar flows. For units involving separation, multiple outlet streams are present. To account for this, eq 23 is utilized:

23 $$\underset{i^{L2}\in I_{j^{L2}}^{Sep,Outlet,Set}}{\sum }S_{i^{L2},c}^{c}=SF_{j^{L2},c}^{1}\underset{i^{L2}\in I_{j^{L2}}^{Sep,Inlet,1,Set}}{\sum }S_{i^{L2},c}^{c}+SF_{j^{L2},c}^{2}\underset{i^{L2}\in I_{j^{L2}}^{Sep,Inlet,2,Set}}{\sum }S_{i^{L2},c}^{c},\forall j^{L2}\in J^{L2},\;c\in C$$

The subset $I_{j^{L2}}^{Sep,Outlet,Set}$ ensures that only one of the outlet streams in a separation unit is included in this equation. $SF_{j^{L2},c}^{1}$ and $SF_{j^{L2},c}^{2}$ are known separation factors in a separation unit for a chemical. These are calculated offline using general methods for calculating the separation of process units. For units such as flash, distillation, VLE separation, and filtration, there is only a single inlet stream; thus, $SF_{j^{L2},c}^{1}$ is active while $SF_{j^{L2},c}^{2}$ is zero. Additionally in this scenario $I_{j^{L2}}^{Sep,Inlet,1,Set}$ ensures that only the inlet stream is included while $I_{j^{L2}}^{,Sep,Inlet,2,Set}$ is inactive. In the situation with two inlets, such as the case of absorption, both $SF_{j^{L2},c}^{1}$ and $SF_{j^{L2},c}^{2}$ are active. Additionally specifically for the case of an absorption unit, the subset $I_{j^{L2}}^{Sep,Inlet,1,Set}$ ensures that only the liquid inlet to the absorption unit is included in the summation while $I_{j^{L2}}^{Sep,Inlet,2,Set}$ ensures that only the vapor inlet to the absorption unit is included in this summation.

To account for the amount of solvent needed based on the amount of vapor entering the absorption unit, we impose in eq 24:

24 $$L_{j^{L2}}^{abs}\underset{i^{L2}\in I_{j^{L2}}^{Sep,Inlet,1,Set}}{\sum }\underset{c\in C}{\sum }S_{i^{L2},c}^{c}=R_{j^{L2}}^{abs}\underset{i^{L2}\in I_{j^{L2}}^{Sep,Inlet,2,Set}}{\sum }\underset{c\in C}{\sum }S_{i^{L2},c}^{c},\forall j^{L2}\in J^{L2}$$

where L^*abs*^ and *R* ^*abs*^ are parameters which control the molar ratio of solvent necessary for vapor entering.

For the stoichiometric yield-based reactions, it is desired that the inlet reaction conditions match as closely as the conditions in the cited literature. To do this, one of the chemicals that is entering a unit is chosen as a reference. The subset $C_{i^{L2}}^{Key,Set}$ ensures that only the chemical that is the reference chemical is included in the summation so that the molar flow rate of reference chemical in a stream $S_{i_{L2}}^{rxn,key}$ can be calculated as follows in eq 25:

25 $$\underset{c\in C_{i^{L2}}^{Key,Set}}{\sum }S_{i^{L2},c}^{c}=S_{i^{L2}}^{rxn,key},\forall i^{L2}\in I^{L2}$$

Using $S_{i^{L2}}^{rxn,key,L2}$ , the required molar flow rate of other chemicals in that stream can be determined as follows in eq 26:

26 $${Ratio}_{i^{L2},c}^{1}\;S_{i^{L2},c}^{c}={Ratio}_{i^{L2},c}^{2}\;S_{i^{L2}}^{rxn,key},\forall i^{L2}\in I^{L2},\;c\in C$$

where the parameters ${Ratio}_{i^{L2},c}^{1}$ and ${Ratio}_{i^{L2},c}^{2}$ , which control the molar ratio of chemical to reference chemical needed in the reactors.

### 3.4 Route Selection

To denote which blocks and modules are activated within the superstructure, three binary variables are introduced, where $y_{j^{L1}}^{L1}$ describes if a block is activated, *Y*_τ_ denotes if a certain route in the superstructure is activated or not, and $y_{j^{L1,\tau }}^{production}$ denotes the matching of blocks and module:

$$y_{j^{L1}}^{L1}=\{\begin{array}{ll} 1 & \mathrm{if}\;\mathrm{block}\;j^{L1}\;\mathrm{is}\;\mathrm{activated}\\ 0 & \mathrm{otherwise} \end{array}$$

$$Y_{\tau }=\{\begin{array}{ll} 1 & \mathrm{if}\;\mathrm{route}\;\tau \;\mathrm{is}\;\mathrm{chosen}\\ 0 & \mathrm{otherwise} \end{array}$$

$$y_{j^{L1},\tau }^{production}=\{\begin{array}{ll} 1 & \mathrm{if}\;\mathrm{block}\;j^{L1}\;\mathrm{in}\;a\;\mathrm{route}\;\tau \;\mathrm{is}\;\mathrm{activated}\\ 0 & \mathrm{otherwise} \end{array}$$

The following logical constraints in eqs 27–29 are imposed to ensure that flow into and out of a unit can occur only when that unit is activated:

27 $$0\leq \underset{i^{L1}\in I_{j^{L1}}^{Inlet,L1}}{\sum }\underset{c\in C}{\sum }B_{i^{L1},j^{L1},c}^{In}\leq F^{\max}y_{j^{L1}}^{L1},\forall j^{L1}\in J^{L1}$$

28 $$0\leq \underset{i^{L1}\in I_{j^{L1}}^{Outlet,L1,Out}}{\sum }\underset{c\in C}{\sum }B_{i^{L1},j^{L1},c}^{Out}\leq F^{\max}y_{j^{L1}}^{L1},\forall j^{L1}\in J^{L1}$$

29 $$0\leq \underset{i^{L1}\in I_{j^{L1}}^{Feed,L1}}{\sum }F_{i^{L1},j^{L1}}^{In,L1}\leq F^{\max}y_{j^{L1}}^{L1},\forall j^{L1}\in J^{L1}$$

where *F* ^max^ is an upper bound denoting the maximum possible flow rate. An inequality constraint in eq 30 is added to relate the activation of a block to that block, along with its route:

30 $$y_{j^{L1}}^{L1}\leq \underset{\tau \in T_{j^{L1}}^{BRM}}{\sum }y_{j^{L1},\tau }^{production},\forall j^{L1}\in J^{L1}$$

Since each route is made up of specific units, a constraint is added to ensure that only blocks belonging to a given route are activated if that route is activated with eq 31:

31 $$y_{j^{L1},\tau }^{production}=Y_{\tau },\forall j^{L1}\in J^{L1},\;\tau \in T_{j^{L1}}^{BRM}$$

Lastly, blocks such as the PMIDA and PMG1 block cannot be utilized for two routes at the same time. This is due to varying cost functions for these blocks based on the composition of the inlet stream. As a result, the following constraint in eq 32 is added along with the subset *T* ^*NM*^, which is the set of route combinations that are exclusive:

32 $$\underset{\tau \in T^{NM}}{\sum }Y_{\tau }\leq 1$$

### 3.5 Capacity Constraints

Each block of the superstructure has its own associated capacity based on the desired output of the block. Therefore, the capacity of each block can be determined based on the total outlet of the key L1 stream of the block $I_{j^{L1}}^{Capacity,Outlets}$ . The capacity of a block *j* ^L1^ is broken down into the different routes τ using $P_{j^{L1},\tau }^{Cap}$ as shown in eq 33:

33 $$\underset{i^{L1}\in I_{j^{L1}}^{Capacity,Outlets}}{\sum }\underset{c\in C}{\sum }B_{i^{L1},j^{L1},c}^{out}=\underset{\tau \in T_{j^{L1}}^{BRM}}{\sum }P_{j^{L1},\tau }^{Cap},\forall j^{L1}\in J^{L1}$$

To ensure that the capacity is only that of the correct block-route pairing, the constraint in eq 34 is added:

34 $$P_{j^{L1},\tau }^{\min}\;y_{j^{L1},\tau }^{production}\leq P_{j^{L1},\tau }^{Cap}\leq P_{j^{L1},\tau }^{\max}\;y_{j^{L1},\tau }^{production},\forall j^{L1}\in J^{L1},\;\tau \in T_{j^{L1}}^{BRM}$$

where parameters $P_{j^{L1},\tau }^{\min}$ and $P_{j^{L1},\tau }^{\max}$ along with $y_{j^{L1},\tau }^{production}$ are utilized to ensure only if the route is chosen that the block-route pairing is chosen.

For either a feed or product storage unit, a storage unit is only chosen if it is used for storage at any time. Therefore, the amount stored at any time $W_{i^{L1},t}^{s}$ and $S_{t}^{s}$ must be less than a capacity *V* ^*Max*^. Parameters ${\left(\rho_{i^{L1}}^{W}\right)}^{-1}$ and ${\left(\rho^{s}\right)}^{-1}$ are conversion factors to convert the moles stored to volume. Binaries $y_{i^{L1}}^{w,storage}$ and *y* ^*s*,*storage*^ are introduced to denote whether a unit is chosen for storage or not, with the following constraints in eqs 35 and 36:

35 $$0\leq {\left(\rho_{i^{L1}}^{W}\right)}^{-1}W_{i^{L1},t}^{s}\leq V^{\mathrm{Max}}y_{i^{L1}}^{w,storage},\forall i^{L1}\in I^{L1,Feed},\;t\in T$$

$$y_{i^{L1}}^{w,storage}=\{\begin{array}{ll} 1 & \mathrm{if}\;\mathrm{feed}\;\mathrm{storage}\;\mathrm{is}\;\mathrm{utilized}\\ 0 & \mathrm{otherwise} \end{array}$$

36 $$0\leq {\left(\rho^{s}\right)}^{-1}S_{t}^{s}\leq V^{\mathrm{Max}}y^{s,storage},\forall t\in T$$

$$y^{s,storage}=\{\begin{array}{ll} 1 & \mathrm{if}\;\mathrm{product}\;\mathrm{storage}\;\mathrm{is}\;\mathrm{utilized}\\ 0 & \mathrm{otherwise} \end{array}$$

An additional constraint is added to the initial storage volume $W_{i^{L1}}^{s,initial}$ as follows:

37 $$0\leq {\left(\rho_{i^{L1}}^{W}\right)}^{-1}W_{i^{L1}}^{s,initial}\leq V^{\mathrm{Max}}y_{i^{L1}}^{w,storage},\forall i^{L1}\in I^{L1,Feed}$$

While eq 37 is in general not necessary since $W_{i^{L1}}^{s,initial}$ and *S* ^*s*,*initial*^ are bounded by the storage at the first period, which is already bounded by the above equations, the additional constraint helps in solving the model.

### 3.6 Objective: Minimizing Process Cost

For any process, the overall goal is not only to satisfy the demand from the customer but also to produce the desired product in a cost-effective manner. Thus, it is desired to minimize the total cost, *TC*, which is given by eq 38:

38 $$TC=\underset{j^{L1}\in J^{L1}}{\sum }\underset{\tau \in T_{j^{L1}}^{BRM}}{\sum }PC_{j^{L1},\tau }+\underset{i^{L1}\in I^{L1,Feed}}{\sum }WC_{i^{L1}}+SC+\underset{i^{L1}\in I^{L1,Feed}}{\sum }\underset{t\in T}{\sum }FC_{i^{L1},t}$$

where the total cost consists of the feed cost $FC_{i^{L1},t}$ , the costs of building and operating each production module utilized in the superstructure $PC_{j^{L1},\tau }$ , and the costs of building feed and product storage units $WC_{i^{L1}}$ and *SC*, respectively. The feed costs can be determined by the amount of feed material $TF_{i^{L1}}$ fed into the feed streams of the superstructure multiplied by the cost factor $FCF_{i^{L1},t}$ of each feed stream at each time step $FCF_{i^{L1,t}}$ as shown in eq 39:

39 $$FC_{i^{L1},t}=FCF_{i^{L1},t}\underset{c}{\sum }MW_{c}\;z_{i^{L1},c}^{L1}\;Feed_{i^{L1},t}^{in},\forall i^{L1}\in I^{L1,Feed},\;t\in T$$

where $FCF_{i^{L1},t}$ is a parameter that contains both the cost per amount and the time step.

Some blocks have multiple blocks that feed into them, such as the PMIDA block. This can impact the costing of the process units in the block, as for example, the inlet from DSIDA1 and DSIDA2 have varying compositions that can impact the costing of units in the PMIDA block. Cost equations as a function of the annual production flow rate of each block were developed using capital cost equations along with the assumption that the cost follows the rule of six-tenths with eq 40:

40 $$PC_{j^{L1},\tau }=A_{j^{L1},\tau }^{PC}{\left[P_{j^{L1},\tau }^{Cap}\right]}^{B_{j^{L1},\tau }^{PC}},\forall j^{L1}\in J^{L1},\;\tau \in T_{j^{L1}}^{BRM}$$

where $A_{j^{L1,\tau }}^{PC}$ and $B_{j^{L1},\tau }^{PC}$ denote plant costing factors for the various blocks.^56–59^ Additionally, to convert the total cost to an annual cost, a cost recovery factor of 0.15 was incorporated into the cost analysis as described in the Supporting Information.

Since the storage capacity is fixed to a single size, the cost of the storage unit is essentially only if the unit is chosen to be built, as shown in eqs 41 and 42:

41 $$WC_{i^{L1}}=SCF\times y_{i^{L1}}^{w,storage},\forall i^{L1}\in I^{L1,Feed}$$

42 $$SC=SCF\times y^{s,storage},\forall i^{L1}\in I^{L1,Feed}$$

where *SCF* is a storage cost factor determined from the costing of storage vessels adjusted to 2022 and with a capital recovery factor of 0.15.^56,59^

Overall, the process synthesis problem is described by a mixed-integer nonlinear program (MINLP) with the objective of minimizing the total cost (*TC*), as given in eq 38, subject to the constraints in eqs 1–42.

## 4 Case Study

We apply the MINLP approach to design a glyphosate plant to meet the year-long variable demand in the San Joaquin Valley in the United States. Due to glyphosate’s usage as a herbicide, the demand for glyphosate is affected by the seasonal variability that affects agricultural production. Based on data from the California Pesticide Information Portal (CalPip), usage of glyphosate in the San Joaquin Valley for the year 2022 varied weekly, with rising usage in the summer weeks.^3^ The San Joaquin Valley consists of eight counties in California and is one of the most agriculturally productive regions in the United States. Over $34 billion in agricultural products are produced in this region each year.^60^ We make the following assumptions:

- Usage of glyphosate reported by CalPip is directly correlated with glyphosate demand.
- Demand for glyphosate is fully realized at a single production facility within the San Joaquin Valley.
- The process is continuous over the entire year; therefore, the weekly demand can be expressed as a flow rate at each time period.
- Demand and prices fluctuate at weekly intervals.
- The cost of transporting feed chemicals to the production facility, along with the transportation of product to customers, is outside the scope of the superstructure optimization of the production facility.

In the absence of data on glyphosate demand, it is assumed that the trend in usage is similar to that of demand. Glyphosate, like many pesticides, can be stored or distributed in either a salt or solution. We use this to estimate two different scenarios, shown in Figure 7. In the first scenario, it is assumed that the glyphosate demand is equivalent to the total product utilized in the given week, represented by the blue bar. This represents a more aggressive estimate of glyphosate demand since we neglect whether glyphosate is in salt or solution form. In the second scenario, we take this consideration into account by assuming the glyphosate demand to be equivalent to the total active ingredient applied, represented by the red bar. As a result, scenario 2 is a more conservative estimate of the actual demand.

**Figure 7:**
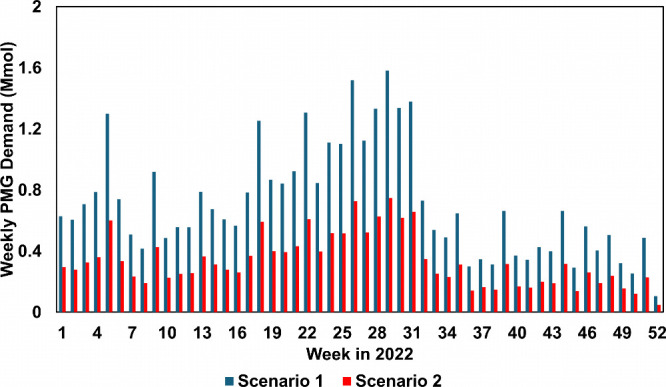
Weekly PMG demand profile in San Joaquin Valley, 2022.

Feed data and pricing were obtained from a variety of available sources in literature, with all the prices utilized given in the Supporting Information^59,61–65^ along with the additional parameters used in the study. The model, which contained 27968 continuous variables, 48 binary variables, and 28191 equations, was solved using SCIP 9.2.0 to optimality in 0.24 s.^66^ The key results from the design are summarized in Table 1.

**Table 1:** Key overall results for the economic design of the two scenarios

	Annual Total Cost ($ ×10^6^)	Weekly Glyphosate Production Capacity (Mmol)	Production Route Chosen	Feed Storage Utilized?	Product Storage Utilized?
Scenario 1	36.825	0.717	DEA	Yes	Yes
Scenario 2	17.208	0.335	DEA	Yes	Yes

Examining the design decisions, the optimal route chosen was the DEA route as shown in Figure 3b. In the DEA route, ethylene oxide is the primary feedstock of interest in comparison to natural gas in the HCN route and a variety of chemicals in the glycine route. Looking at solely the plant costs functions, one would assume that this solution is trivial, as the sum of the plant costs for producing DEA is lower than the other two routes. However, it should be noted that the plant costs due to being annualized through the cost recovery factor make up a small fraction of the overall costs. As a result, the model is highly sensitive to the feed cost profiles. For example, if the cost of ethylene oxide increased by 50% and the cost of formaldehyde, a common chemical in all three routes, decreased by 20%, the HCN route from Figure 3a would be chosen as the optimal route choice. Uncertainty in pricing is inherently present due to volatility as a result of regionality, availability of chemicals, supply chain limitations, etc. This demonstrates the importance of taking account of temporal variabilities in the design of glyphosate production through the superstructure model.

Examining the operational decisions, for the economic design, upstream feed storage is utilized. Due to the varying feed costs, to take advantage of low prices while simultaneously avoiding high prices, excess feed is bought when prices are low and then utilized throughout the year. In Figure 8c for chemicals stored such as ammonia, formaldehyde, ethylene oxide, and others, for the majority of the year, there are little to no chemicals purchased. The brief spikes correlate to the lower range of prices for these feed chemicals. These spikes may come at different time periods due to the individual markets for each chemical. For example, normalized chlorine prices are lowest early in the year, while for ammonia, the normalized prices reach their lowest around week 40. In Figure 8c, chlorine is purchased in bulk early in the year at week 4, while ammonia is purchased in bulk at week 36. As a result of this, the feed storage profile can be obtained as demonstrated in Figure 8a.

**Figure 8:**
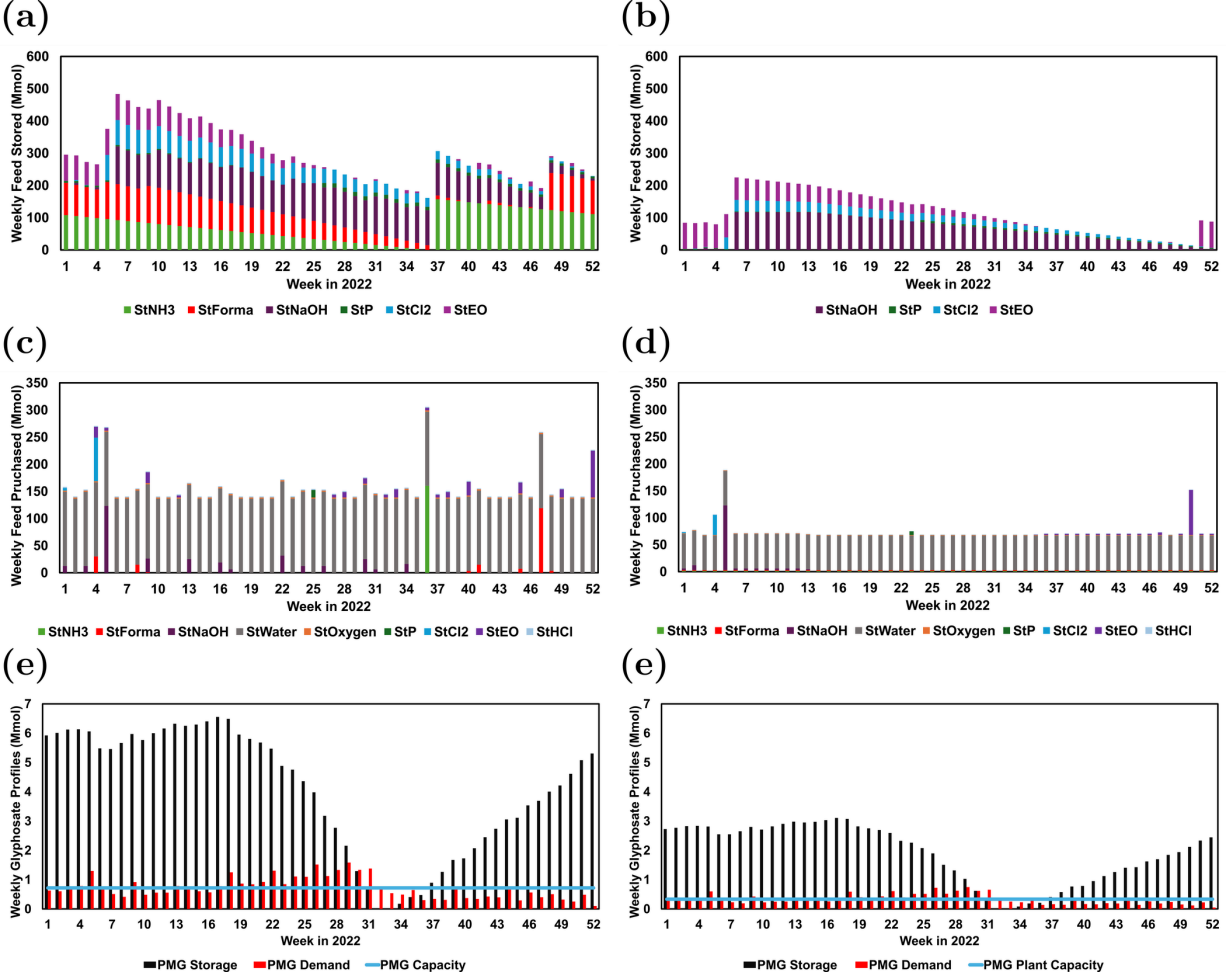
Results for the case study: (a) optimal weekly feed storage for scenario 1; (b) optimal weekly feed storage for scenario 2; (c) optimal weekly feed purchasing for scenario 1; (d) optimal weekly feed purchasing for scenario 2; (e) optimal PMG profiles for scenario 1; (f) optimal PMG profiles for scenario 2.

While not shown directly in Figure 8a, the cyclic constraint in Section 3 enforces that the amount stored at the time step after week 52 exactly matches what was stored in week 1, ensuring that there is some feed stored at the beginning of the next year of operation. It is important to note that even though all chemicals have varying feed prices over the year, not all chemicals are chosen to be stored. Specifically, water, liquid oxygen, air, and hydrochloric acid are chosen not to be stored. This can be attributed to a combination of potential reasons, such as the trade-off between the cost of storage units versus the cost of mitigating price variability, and potentially for gaseous species, the lack of ability to store sufficient inventory as a result of low densities.

Table 2 displays the overall weekly capacities of the main outlet of each process block for glyphosate production in the DEA route. A key observation is that the weekly capacity of glyphosate that is produced is 0.717 Mmol. Relative to this capacity and the demand for glyphosate, the amount of glyphosate stored weekly is extremely high in Figure 8e. While the amount that the demand is higher than the plant capacity is not large, there are extended time periods where capacity does not meet demand, especially between weeks 17 and 31. As a result of this, there needs to be a large buffer of storage to satisfy the demand during all time periods.

**Table 2:** Weekly Process Capacities (in Mmol) for the Economic Design of the Two Scenarios

	DEA	DSIDA2	PCl3	PMIDA	PMG1
Scenario 1	0.922	1.359	1.142	125.663	0.717
Scenario 2	0.431	0.635	0.533	58.668	0.335

The second scenario has the same number of continuous variables, binary variables, and equations and is solved in 0.90 s in SCIP 9.2.0. Key overall results between the two scenarios are shown in Table 1. It is apparent that the high-level design decisions in the two scenarios are extremely similar. In both scenarios, the DEA route is chosen along with upstream storage and downstream glyphosate storage. To see the sensitivity of the overall design to the demand variability, the operational decisions are further examined.

Figure 8a-f shows that the change in demand has a significant impact on the operational scheduling upstream and downstream of the production process. Examining upstream, unlike in the first scenario, liquid ammonia and formaldehyde are not chosen to be stored in Figure 8b. As a result, both ammonia and formaldehyde have a constant purchasing profile. This is due to a trade-off between investment and operating costs in decision-making. To take advantage of varying feed costs, a storage vessel must be purchased and built. As a result, this means that for lower demands of glyphosate, the cost of building the storage vessel outweighs the potential gains from varying feed prices. Additionally, for the similar chemicals stored between the two scenarios, there are differences in the two profiles. In Figure 8e compared to Figure 8f, while ethylene oxide is purchased at many weeks in the later half of the year in the first scenario, in the second, ethylene oxide is purchased in bulk in week 50. This demonstrates that variability in the demand can play a major role in the optimal operational decisions.

Returning to the discussion regarding sensitivity to feed pricing, if the model is solved with a 20% reduction in the price of formaldehyde and a 50% increase in the price of ethylene oxide as done previously, the overall design decision for the process is the DEA route. This is different from what was reported for the first scenario under the same feed pricing change, where the HCN route was chosen. This demonstrates that both the feed pricing and demand variability play major roles in the overall design of the chemical process. Since demand and pricing can vary depending on region, year, and other external disturbances, determining the overall optimal design is a complex relationship between the trade-offs between chemical routes, but also between purchasing versus storage. To capture these complex relationships in a systematic manner, a systems engineering approach such as a superstructure optimization model is necessary.

## 5 Conclusions

Systematic approaches to designing chemical processes are growing in demand to handle the rapidly increasing demand for chemicals globally. For industries with temporal variability in the demand and pricing of raw materials, this is especially important as determining the optimal design and scheduling is complex. Additionally, for some industries such as the pesticide industry, there has been limited work in the literature examining how to systematically design chemical processes under both short-term and seasonal variations in pesticide demands and chemical prices. To address this, we introduced a superstructure modeling approach that can be used to design optimal chemical processes that can be adjusted based on the fidelity of the models. This results in a mixed-integer nonlinear programming (MINLP) model that considers investment and operational costs in the optimization problem. We demonstrated the applicability of the approach by examining the production of glyphosate in the San Joaquin Valley. Two scenarios were examined: one with a demand profile matching the total product applied and the second with a demand profile matching the total chemical applied. Results demonstrate that the varying demand profile impacts the overall design of the process, not only on the choice of chemical route but also on the upstream and downstream storage profiles. Additionally, examining the impact of feed pricing illustrated that the trade-offs in decisions to determine the optimal designs are complex and require a holistic understanding of the overall process.

Results indicate the importance of both upstream and downstream storage to mitigate variabilities. However, storage of hazardous products could substantially increase the process safety risks involved for a process. Future work will examine how to design chemical processes to have both safety and economic considerations under short-term and long-term variabilities.

## Supplementary Material



## Notes

Details on the nomenclature, superstructure block simulations, cost analysis, and data used for the case study (PDF)
