# Supplementary material for: Optimal Process Synthesis of Pesticide Production Considering Variable Demands and Raw Material Prices
Source: Ind Eng Chem Res. 2025 Oct 13;64(43):20692–705. doi: 10.1021/acs.iecr.5c02394 (PMC12576779; doi:10.1021/acs.iecr.5c02394)
Supplement: Supplementary file 1 [file ie5c02394_si_001.pdf]

Optimal Process Synthesis of Pesticide Production Considering Variable Demands and Raw Material Prices

Austin Johnes[1,2], Faisal Khan[1,2], and M. M. Faruque Hasan[1,3*]
[1]Artie McFerrin Department of Chemical Engineering, Texas A&M University
[2]Mary Kay O'Connor Process Safety Center, Texas A&M University
[3]Texas A&M Energy Institute, Texas A&M University
College Station, TX 77843-3122, USA.

Supporting Information

Contents

S1 Nomenclature **2**

S2 Superstructure Blocks Process Simulations **4**

S3 Cost Analysis Data **5**

S4 Parameters **7**

*Correspondence should be addressed to M.M. Faruque Hasan at hasan@tamu.edu, Phone: (979) 862-1449.

S1 Nomenclature

S1.1 Sets and Subsets

Set	Description
I	Set of streams (i)
J	Set of blocks/units (j)
C	Set of chemicals (c)
$\mathcal{T}$	Set of technology routes (τ)
T	Set of times-steps (t)
**Subset**	**Description**
I^{L1}	Subset of streams in the L1 structure (i^{L1})
J^{L1}	Subset of blocks in the L1 structure (j^{L1})
$I^{L1,Feed}$	Feed streams in the L1
$I_{j^{L1}}^{Feed,L1}$	L2 feed stream with L2 block
$I^{L1,Process}$	Process streams in the L1
$I_{j^{L1}}^{Inlet,L1}$	L1 Inlet process streams for L1 block
$I_{j^{L1}}^{Outlet,L1,Out}$	L1 Outlet process streams for L1 block
$I_{i^{L1},j^{L1}}^{Feed,L1,L2}$	Connection of L2 feed streams with L1 feed streams and L1 block
$I_{i^{L1},j^{L1}}^{Process,Out,L1,L2}$	Connection of L2 outlet streams with L1 outlet streams and L2 unit
$I_{i^{L1},j^{L1}}^{Process,In,L1,L2}$	Connection of L2 inlet streams with L1 inlet streams and L2 unit
$I_{j^{L2}}^{Feed,In,Set}$	L2 feed stream with L2 unit
$I_{j^{L2}}^{Stream,In,Set}$	L2 inlet process stream with L2 unit
$I_{j^{L2}}^{Stream,Out,Set}$	L2 outlet process stream with L2 unit
$I_{j^{L2}}^{Sep,Inlet,1,Set}$	L2 inlet process stream with L2 separation unit
$I_{j^{L2}}^{Sep,Inlet,2,Set}$	L2 inlet process stream with L2 separation unit
$I_{j^{L2}}^{Sep,Outlet,Set}$	L2 outlet process stream with L2 separation unit
$I_{j^{L1}}^{Capacity,Outlets}$	L1 main outlet streams
$J_{i^{L1}}^{Feed,L1}$	L1 block with L1 feed stream
$J_{i^{L1}}^{Outlet,L1}$	L1 blocks of L1 process outlet streams
$J_{i^{L1}}^{Inlet,L1}$	L1 blocks of L1 process inlet streams
$J_{i^{L1}}^{L1,Product,Match}$	L1 blocks of L1 process outlet streams with main
$J^{L2,Rxn}$	L2 reaction unit
$J^{L2,No,Rxn}$	L2 unit that is not a reaction unit
$C^{Product}$	Chemical product of interest
$C_{j^{L2}}^{LR,Set}$	Limiting reagent chemical of L2 unit
$C_{i^{L2}}^{Key,Set}$	Reference chemical of L2 inlet reaction stream
$\mathcal{T}^{NM}$	Technologies that cannot be used together
$\mathcal{T}_{j^{L1}}^{BRM}$	Matching of L1 blocks to technology routes

S1.2 Variables

**Binary Variable**	**Description**
$y_{j^{L1}}^{L1}$	Choice of L1 block
$y_{j^{L1},\tau}^{production}$	Choice of L1 block with route
Y_{τ}	Choice of production route
$y_{i^{L1}}^{w,storage}$	Choice of upstream feed storage
$y^{s,storage}$	Choice of downstream product storage
**Continuous Variable**	**Description**
$Feed_{i^{L1},t}^{In}$	Flowrate of feed into the storage system ($\frac{Mmol}{week}$)
$TF_{i^{L1}}$	Flowrate of feed out of the storage system into superstructure ($\frac{Mmol}{week}$)
$W_{i^{L1},t}^{s}$	Feed stored ($Mmol$)
$W_{i^{L1}}^{initial}$	Feed stored before and after time-period ($Mmol$)
$TF_{i^{L1},j^{L1}}^{in,L1}$	Flowrate of L1 feed stream into L1 block ($\frac{Mmol}{week}$)
$B_{i^{L1},j^{L1},c}^{In}$	Flowrate of a chemical in L1 process inlet stream into L1 block ($\frac{Mmol}{week}$)
$B_{i^{L1},j^{L1},c}^{Out,}$	Flowrate of a chemical in L1 process outlet stream out of L1 block ($\frac{Mmol}{week}$)
S_{t}^{s}	Product stored ($Mmol$)
$S^{s,initial}$	Product stored before and after time-period ($Mmol$)
P_{t}^{out}	Product flowrate out of storage system ($\frac{Mmol}{week}$)
$F_{i^{L2}}$	Flowrate in L2 feed stream ($\frac{Mmol}{week}$)
$S_{i^{L2},c}^{c}$	Flowrate of a chemical in L1 process stream ($\frac{Mmol}{week}$)
$F_{j^{L2},c}^{in}$	Feed flowrate of a chemical into L2 unit ($\frac{Mg}{week}$)
$S_{j^{L2},c}^{in}$	Process flowrate of a chemical into L2 unit ($\frac{Mg}{week}$)
$S_{j^{L2},c}^{out}$	Process flowrate of a chemical out of L2 unit ($\frac{Mg}{week}$)
$LR_{j^{L2}}$	Limiting reagent flowrate into L2 unit ($\frac{Mmol}{week}$)
$R_{j^{L2},c}$	Flowrate produced or reacted of a chemical in the L2 unit ($\frac{Mg}{week}$)
$S_{i^{L2}}^{rxn,key}$	Reference chemical flowrate of a L2 reactor inlet stream ($\frac{Mmol}{week}$)
$P_{j^{L1},\tau}^{cap}$	L1 block capacity of a technology route ($\frac{Mmol}{week}$)
TC	Total annual cost of system (\$)
$PC_{j^{L1},\tau}$	L1 block annual cost of a technology route (\$)
$WC_{i^{L1}}$	Storage unit of chemical annual cost (\$)
SC	Storage unit of product annual cost (\$)
$FC_{i^{L1},t}$	Weekly cost of feed chemical (\$)

S1.3 Parameters

Parameter	Description
$\chi_{j^{L2},c}$	Chemical conversion factor in L2 reaction unit
$SF^1_{j^{L2},c}$	Vapor separation factor for inlet (liquid inlet for absorption) in L2 separation unit
$SF^2_{j^{L2},c}$	Vapor separation factor for vapor inlet in L2 absorption unit
$L^{abs}_{j^{L2}}$	Denominator of solvent to gas ratio
$R^{abs}_{j^{L2}}$	Numerator of solvent to gas ratio
$Ratio^1_{i^{L2},c}$	Denominator of chemical to reference chemical ratio
$Ratio^2_{i^{L2},c}$	Numerator of chemical to reference chemical ratio
$z_{i^{L2},c}$	L2 feed stream composition
$FCF_{i^{L1},t}$	L1 feed cost factor ($\frac{\$.Week}{Mg}$)
$z^{L1}_{i^{L1},c}$	L1 feed stream composition
$P^{min}_{j^{L1},\tau}$	L1 block minimum capacity ($\frac{Mmol}{Week}$)
$P^{max}_{j^{L1},\tau}$	L1 block maximum capacity ($\frac{Mmol}{Week}$)
$A^{PC}_{j^{L1},\tau}$	L1 block plant cost factor
$B^{PC}_{j^{L1},\tau}$	L1 block plant cost factor
$(\rho^W_{i^{L1},c})^{-1}$	Inverse molar density of L1 feed ($\frac{cm^3}{mol}$)
$(\rho^s)^{-1}$	Inverse molar density of product ($\frac{cm^3}{mol}$)
V^{max}	Max storage capacity (m^3)
SCF	Annual storage cost factor (\$)
ΔT	Time-step ($Week$)
F^{max}	Big-M max flowrate ($\frac{Mmol}{Week}$)
$Demand_t$	Demand flowrate ($\frac{Mmol}{Week}$)
MW_c	Molecular weight of chemical ($\frac{g}{mol}$)

S2 Superstructure Blocks Process Simulations

To develop the individual blocks of superstructure, literature data available and general methods for process separations were utilized. Mass flow and composition data needed for obtaining both cost functions were obtained from a simulation model similar to that described in the L2 superstructure. Instead of using blocks for superstructures, simulations were performed to produce glyphosate for each route. Results of these simulations were checked against simulations of the superstructure blocks to verify result accuracy. Several approximations were made to determine the unknown pressure and temperature data. For stream mixing, it was approximated that isothermal mixing occurs. Since the flowsheets were designed so that each inlet stream to a mixer had the same temperature, it is approximated based on isothermal mixing that the outlet stream has the same temperature. For the outlet temperature of pumps, it was assumed that the temperature change across the pump is negligible, and thus the temperature of the outlet is the same as the temperature of the inlet. For distillation and absorption towers, the outlet streams are either at the dew point or the bubble point. Using the composition data along with saturated pressure data for each chemical, the outlet temperatures were estimated in Aspen HYSYS. Additionally, any heat exchanger where the outlet temperature is unknown but complete phase change occurs is also estimated in Aspen using composition data obtained from the simulations[1]. For some flash tanks and VLE separators, the outlet pressure was determined based on the fixed temperature and

recovery of the key component through an iterative process. Due to a lack of data on filtration units, it was assumed that the separation of glyphosate through this unit from the solution was 98%.

S3 Cost Analysis Data

While the majority of chemical property data needed for calculations was obtained from Aspen HYSYS, additional data was retrieved from literature for comprehensive calculations as follows:

1. Saturated Vapor Pressure Data[1–5]
2. Liquid/Solution Density Data[6–9]
3. Heat of Formation, Vaporization, Critical Temperature, and Boiling Point Data[7,10–12]
4. Heat of Fusion Data[13]

For chemicals where little to zero property data, such as PMIDA and glyphosate, is available in literature, parameter estimation techniques were utilized based as well[14–19]. To develop a surrogate model for the plant cost as a function of the capacity, a detailed scale-up of each flowsheet was performed at each of the simulation points. This included the sizing of the reactor, flash vessels, separation vessels, distillation column, absorption columns, heat exchangers, compressors, turbines, and pumps[20–22]. For heat calculations, Watson's Correlation was used to estimate the enthalpy of vaporization at various temperatures[23]. It should be noted that filtration units were not sized, as information regarding particle size was not available.

Once sizing was completed, a reference capacity for economic analysis was determined. This reference capacity was chosen such that it represents the acceptable ranges of the cost correlations. The cost at the reference point from the sources[20,21] was then adjusted to 2022 (using the chemical engineering plant cost index, CEPCI value of 816[24]) and annualized using a cost recovery factor of 0.15. The cost recovery factor 0.15 was estimated based on a thirty-year lifetime of operation and a roughly 15% interest rate. The rule of six-tenths was then applied to estimate the cost as a function of the capacity[25].Data for the base reference capacity and costs of each block are shown in Table S1. Data used in the process synthesis model is shown in Tables S2 - S16.

Table S1: Reference Capacities and Costs for Each Block.

j^{L1}	τ	Reference Capacity ($\frac{Mmol}{week}$)	Reference Annual Cost ($)
B_PCl3	P_HCN	3.881	2.541e+4
B_HCN	P_HCN	11.747	6.204e+5
B_IDAN	P_HCN	15.198	1.021e+6
B_DSIDA1	P_HCN	115.617	9.499e+5
B_PMIDA	P_HCN	429.029	2.430e+5
B_PMG1	P_HCN	2.437	4.705e+4
B_PCl3	P_DEA	3.881	2.541e+4
B_DEA	P_DEA	3.135	1.406e+5
B_DSIDA2	P_DEA	4.620	2.526e+4
B_PMIDA	P_DEA	427.147	4.239e+4
B_PMG1	P_DEA	2.437	1.169e+5
B_PCl3	P_Glycine	3.316	2.391e+4
B_DEPP	P_Glycine	2.593	4.100e+4
B_Glycine	P_Glycine	20.022	6.715e+4
B_PMG2	P_Glycine	2.437	2.719e+5

S4 Parameters

Table S2: Storage Units General Parameters.

V^{max} (m^3)	WCF (\$)	SCF (\$)
4000	1.898e+5	1.898e+5

Table S3: Absorption Ratio Parameters.

j^{L2}	$L^{abs}_{j^{L2}}$	$R^{abs}_{j^{L2}}$
S1_1	1	6.773

Table S4: General Parameters.

ΔT	F^{max} (Mmol/Week)
1	1.000e+3

Table S5: Vapor Separation Factor $SF^1_{j^{L2},c}$ Part 1 (Liquid for Most Cases, VLE for Separator Unit).

	Acetic_Acid	Acetic_Anhydride	Aminoacetonitrile	Ammonia	Ammonium_Chloride	CO2	Chlorine	DCA	DEA	DEP	DSIDA	EO	Ethanol	Ethyl_Chloride	Formaldehyde	Glycine	HCN
S1_1	3.214e-2	1.435e-2	7.723e-3	1.000e+0	2.124e-6	1.000e+0	1.000e+0	1.130e-3	1.377e-5	5.532e-3	1.821e-7	9.787e-1	1.271e-1	9.270e-1	1.000e+0	1.046e-8	7.136e-1
S1_2	4.325e-1	3.069e-1	2.588e-1	9.671e-1	1.670e-3	9.879e-1	9.385e-1	1.042e-1	1.065e-2	1.085e-1	3.664e-4	8.821e-1	7.433e-1	8.602e-1	9.309e-1	5.115e-5	8.746e-1
S1_3	3.977e-1	2.270e-1	1.359e-1	9.911e-1	4.206e-5	9.981e-1	9.867e-1	2.228e-2	2.713e-4	1.022e-1	3.568e-6	9.584e-1	7.230e-1	9.528e-1	9.819e-1	2.030e-7	9.369e-1
S1_4	8.645e-1	7.665e-1	6.789e-1	9.981e-1	1.876e-3	9.995e-1	9.969e-1	3.105e-1	1.349e-2	5.080e-1	2.422e-4	9.919e-1	9.620e-1	9.905e-1	9.960e-1	2.048e-5	9.890e-1
S1_5	0.000e+0	0.000e+0	0.000e+0	0.000e+0	0.000e+0	0.000e+0	0.000e+0	0.000e+0	0.000e+0	0.000e+0	0.000e+0	0.000e+0	0.000e+0	0.000e+0	0.000e+0	0.000e+0	0.000e+0
S2_1	5.214e-6	3.401e-8	6.123e-10	1.000e+0	1.241e-31	1.000e+0	1.000e+0	3.740e-15	3.762e-27	2.453e-10	3.294e-38	9.999e-1	1.411e-2	9.999e-1	1.000e+0	9.099e-46	9.990e-1
S2_2	8.547e-4	1.073e-4	4.319e-5	9.990e-1	5.817e-14	1.000e+0	9.878e-1	6.454e-7	7.186e-11	7.675e-7	1.748e-16	8.418e-1	1.237e-1	7.158e-1	9.806e-1	9.366e-20	8.029e-1
S2_3	6.855e-1	2.716e-3	8.994e-6	1.000e+0	1.822e-32	1.000e+0	1.000e+0	1.270e-12	1.714e-28	1.000e-4	8.057e-41	1.000e+0	9.999e-1	1.000e+0	1.000e+0	3.519e-50	1.000e+0
S3_1	1.870e-2	9.189e-3	5.523e-3	6.780e-1	2.992e-6	8.974e-1	5.696e-1	1.013e-3	2.115e-5	3.134e-3	3.252e-7	3.175e-1	7.032e-2	2.855e-1	5.007e-1	2.341e-8	2.437e-1
S3_2	1.000e+0	1.000e+0	9.999e-1	1.000e+0	2.911e-6	1.000e+0	1.000e+0	9.905e-1	1.000e-3	9.997e-1	3.861e-9	1.000e+0	1.000e+0	1.000e+0	1.000e+0	1.494e-12	1.000e+0
S3_3	3.374e-1	1.986e-1	1.292e-1	9.825e-1	7.993e-5	9.957e-1	9.725e-1	2.638e-2	5.649e-4	7.749e-2	8.691e-6	9.256e-1	6.690e-1	9.144e-1	9.640e-1	6.254e-7	8.959e-1
S4_1	7.301e-1	5.683e-1	4.408e-1	9.967e-1	4.245e-4	9.992e-1	9.947e-1	1.258e-1	2.994e-3	3.086e-1	4.617e-5	9.851e-1	9.148e-1	9.827e-1	9.930e-1	3.322e-6	9.786e-1
S4_2	1.000e+0	1.000e+0	1.000e+0	1.000e+0	1.135e-2	1.000e+0	1.000e+0	1.000e+0	9.990e-1	1.000e+0	2.067e-6	1.000e+0	1.000e+0	1.000e+0	1.000e+0	1.551e-11	1.000e+0
S4_3	1.000e+0	9.999e-1	9.905e-1	1.000e+0	7.903e-28	1.000e+0	1.000e+0	2.737e-5	2.918e-20	3.949e-1	2.056e-36	1.000e+0	1.000e+0	1.000e+0	1.000e+0	1.354e-46	1.000e+0
S5_1	1.614e-2	5.464e-3	2.137e-3	9.000e-1	1.045e-7	9.837e-1	8.774e-1	1.632e-4	4.192e-7	3.441e-3	4.598e-9	6.065e-1	5.849e-2	5.859e-1	8.211e-1	1.407e-10	4.621e-1
S5_2	2.006e-1	1.225e-1	9.191e-2	9.314e-1	2.246e-4	9.774e-1	8.824e-1	2.710e-2	1.590e-3	3.845e-2	3.890e-5	7.663e-1	4.949e-1	7.307e-1	8.619e-1	4.348e-6	7.300e-1
S5_3	5.370e-2	2.323e-2	1.174e-2	9.252e-1	2.116e-6	9.844e-1	8.961e-1	1.489e-3	1.259e-5	1.004e-2	1.545e-7	7.119e-1	1.823e-1	6.864e-1	8.596e-1	7.634e-9	6.051e-1
S6_1	8.411e-1	6.985e-1	5.489e-1	9.990e-1	2.904e-4	9.998e-1	9.985e-1	1.449e-1	1.833e-3	4.795e-1	2.363e-5	9.949e-1	9.543e-1	9.942e-1	9.979e-1	1.293e-6	9.921e-1
S6_2	9.516e-1	9.162e-1	8.880e-1	9.991e-1	1.730e-2	9.997e-1	9.983e-1	6.859e-1	1.110e-1	7.581e-1	3.039e-3	9.961e-1	9.871e-1	9.953e-1	9.980e-1	3.407e-4	9.953e-1
S6_3	0.000e+0	0.000e+0	0.000e+0	0.000e+0	0.000e+0	0.000e+0	0.000e+0	0.000e+0	0.000e+0	0.000e+0	0.000e+0	0.000e+0	0.000e+0	0.000e+0	0.000e+0	0.000e+0	0.000e+0
Sp1_1	6.000e-1	6.000e-1	6.000e-1	6.000e-1	6.000e-1	6.000e-1	6.000e-1	6.000e-1	6.000e-1	6.000e-1	6.000e-1	6.000e-1	6.000e-1	6.000e-1	6.000e-1	6.000e-1	6.000e-1

Table S6: Vapor Separation Factor $SF^1_{j^{L2},c}$ Part 2 (Liquid for Most Cases, VLE for Separator Unit).

	HCl	Hydrogen	IDAN	MCA	MEA	Methane	Methanol	NaCl	NaOH	Nitrogen	Oxygen	PCL3	PMG	PMIDA	Paraformaldehyde	Phosphoric_Acid	Phosphorus	TEA	Triethylamine	Water
S1_1	1.000e+0	1.000e+0	5.943e-5	1.305e-3	2.403e-3	1.000e+0	2.158e-1	0.000e+0	3.184e-16	1.000e+0	1.000e+0	1.401e-1	8.203e-14	0.000e+0	1.000e+0	4.916e-7	6.064e-4	2.772e-7	9.400e-2	5.518e-2
S1_2	9.849e-1	9.993e-1	1.994e-2	1.173e-1	1.923e-1	9.929e-1	7.974e-1	3.263e-17	7.276e-12	9.959e-1	9.957e-1	5.981e-1	5.591e-14	3.913e-19	9.309e-1	3.062e-4	2.235e-2	9.842e-4	5.395e-1	6.021e-1
S1_3	9.975e-1	1.000e+0	1.181e-3	2.564e-2	4.621e-2	9.995e-1	8.164e-1	1.512e-23	6.077e-15	9.998e-1	9.997e-1	7.440e-1	1.754e-12	1.228e-17	9.819e-1	9.758e-6	1.226e-2	5.366e-6	6.605e-1	5.308e-1
S1_4	9.994e-1	1.000e+0	3.928e-2	3.423e-1	4.890e-1	9.998e-1	9.742e-1	8.272e-20	1.219e-12	9.999e-1	9.999e-1	9.502e-1	3.378e-12	2.365e-17	9.960e-1	3.926e-4	1.251e-1	5.475e-4	9.339e-1	9.216e-1
S1_5	0.000e+0	0.000e+0	0.000e+0	0.000e+0	0.000e+0	0.000e+0	0.000e+0	0.000e+0	0.000e+0	0.000e+0	0.000e+0	0.000e+0	9.800e-1	0.000e+0	0.000e+0	0.000e+0	0.000e+0	0.000e+0	0.000e+0	0.000e+0
S2_1	1.000e+0	1.000e+0	4.416e-23	8.523e-15	2.878e-13	1.000e+0	2.989e-1	1.115e-142	6.395e-90	1.000e+0	1.000e+0	5.934e-2	1.656e-71	2.464e-101	1.000e+0	3.282e-35	4.014e-16	1.139e-37	5.084e-3	1.000e-4
S2_2	1.000e+0	1.000e+0	8.206e-10	1.075e-6	1.000e-5	1.000e+0	3.135e-1	2.136e-66	6.090e-46	1.000e+0	1.000e+0	1.095e-2	4.985e-54	9.597e-74	9.806e-1	8.792e-17	1.282e-9	7.669e-15	4.409e-3	1.165e-2
S2_3	1.000e+0	1.000e+0	1.282e-22	4.164e-12	1.258e-10	1.000e+0	1.000e+0	4.159e-167	3.172e-100	1.000e+0	1.000e+0	1.000e+0	6.263e-67	2.055e-99	1.000e+0	4.594e-36	1.033e-11	4.154e-42	1.000e+0	9.689e-1
S3_1	8.694e-1	9.979e-1	7.359e-5	1.171e-3	2.156e-3	9.629e-1	1.061e-1	1.863e-23	1.053e-15	9.820e-1	9.797e-1	6.234e-2	1.931e-14	1.352e-19	5.007e-1	6.522e-7	3.949e-4	6.391e-7	4.522e-2	3.299e-2
S3_2	1.000e+0	1.000e+0	3.978e-2	9.938e-1	9.990e-1	1.000e+0	1.000e+0	1.236e-57	1.732e-34	1.000e+0	1.000e+0	1.000e+0	1.025e-30	4.161e-46	1.000e+0	3.084e-8	8.622e-1	2.902e-8	1.000e+0	1.000e+0
S3_3	9.944e-1	9.999e-1	1.963e-3	3.039e-2	5.459e-2	9.986e-1	7.603e-1	4.979e-22	2.814e-14	9.993e-1	9.992e-1	6.398e-1	5.161e-13	3.613e-18	9.640e-1	1.743e-5	1.045e-2	1.708e-5	5.586e-1	4.769e-1
S4_1	9.989e-1	1.000e+0	1.034e-2	1.427e-1	2.347e-1	9.997e-1	9.440e-1	2.645e-21	1.495e-13	9.999e-1	9.999e-1	9.042e-1	2.742e-12	1.919e-17	9.930e-1	9.258e-5	5.310e-2	9.072e-5	8.705e-1	8.288e-1
S4_2	1.000e+0	1.000e+0	9.999e-1	1.000e+0	1.000e+0	1.000e+0	1.000e+0	2.278e-87	7.560e-57	1.000e+0	1.000e+0	1.000e+0	2.094e-79	1.937e-114	1.000e+0	7.308e-8	9.996e-1	1.000e-3	1.000e+0	1.000e+0
S4_3	1.000e+0	1.000e+0	1.946e-15	1.000e-4	2.260e-2	1.000e+0	1.000e+0	4.215e-181	4.834e-112	1.000e+0	1.000e+0	1.000e+0	8.684e-101	1.041e-146	1.000e+0	1.012e-33	6.169e-9	8.441e-34	1.000e+0	1.000e+0
S5_1	9.779e-1	9.999e-1	3.940e-6	2.004e-4	3.368e-4	9.973e-1	1.195e-1	1.994e-29	1.432e-18	9.990e-1	9.987e-1	1.189e-1	6.038e-13	4.226e-18	8.211e-1	2.856e-8	2.581e-4	2.531e-9	7.559e-2	2.404e-2
S5_2	9.714e-1	9.991e-1	3.665e-3	3.105e-2	5.516e-2	9.889e-1	5.778e-1	2.950e-19	4.206e-13	9.939e-1	9.935e-1	3.688e-1	4.382e-14	3.067e-19	8.619e-1	4.359e-5	6.522e-3	1.022e-4	3.118e-1	3.259e-1
S5_3	9.791e-1	9.999e-1	6.359e-5	1.716e-3	3.149e-3	9.961e-1	2.861e-1	1.361e-25	1.786e-16	9.984e-1	9.980e-1	2.236e-1	2.718e-13	1.903e-18	8.596e-1	5.096e-7	1.013e-3	1.915e-7	1.572e-1	8.638e-2
S6_1	9.997e-1	1.000e+0	8.247e-3	1.637e-1	2.648e-1	9.999e-1	9.731e-1	6.478e-23	3.615e-14	1.000e+0	1.000e+0	9.604e-1	1.655e-11	1.158e-16	9.979e-1	6.808e-5	8.929e-2	3.376e-5	9.414e-1	9.000e-1
S6_2	9.996e-1	1.000e+0	2.238e-1	7.152e-1	8.206e-1	9.999e-1	9.908e-1	2.312e-17	3.296e-11	9.999e-1	9.999e-1	9.786e-1	3.434e-12	2.404e-17	9.980e-1	3.405e-3	3.397e-1	7.944e-3	9.726e-1	9.743e-1
S6_3	0.000e+0	0.000e+0	0.000e+0	0.000e+0	0.000e+0	0.000e+0	0.000e+0	0.000e+0	0.000e+0	0.000e+0	0.000e+0	0.000e+0	9.800e-1	0.000e+0	0.000e+0	0.000e+0	0.000e+0	0.000e+0	0.000e+0	0.000e+0
Sp1_1	6.000e-1	6.000e-1	6.000e-1	6.000e-1	6.000e-1	6.000e-1	6.000e-1	6.000e-1	6.000e-1	6.000e-1	6.000e-1	6.000e-1	6.000e-1	6.000e-1	6.000e-1	6.000e-1	6.000e-1	6.000e-1	6.000e-1	6.000e-1

Table S7: Vapor Separation Factor from Vapor Inlet for Absorption Tower $SF^2_{j^{L2},c}$.

j^{L2}	c	$S2^1_{j^{L2},c}$
S1_1	Water	6.594e-22
S1_1	Ammonia	8.113e-1
S1_1	Formaldehyde	6.133e-1
S1_1	NaOH	2.694e-261
S1_1	HCl	9.465e-1
S1_1	NaCl	0.000e+0
S1_1	phosphorus	8.026e-55
S1_1	Chlorine	7.180e-1
S1_1	PCL3	3.839e-15
S1_1	PMG	9.320e-221
S1_1	Methanol	4.973e-12
S1_1	Ethanol	7.527e-16
S1_1	Methane	9.888e-1
S1_1	HCN	1.000e-3
S1_1	EO	1.144e-1
S1_1	MEA	9.074e-45
S1_1	DEA	1.854e-82
S1_1	TEA	5.581e-111
S1_1	IDAN	8.774e-72
S1_1	DSIDA	4.742e-114
S1_1	PMIDA	0.000e+0

S1_1	Hydrogen	9.995e-1
S1_1	Oxygen	9.942e-1
S1_1	CO2	9.599e-1
S1_1	Ethyl_Chloride	4.193e-2
S1_1	DEP	1.111e-38
S1_1	Acetic_Anhydride	1.008e-31
S1_1	Acetic_Acid	7.650e-26
S1_1	MCA	3.171e-49
S1_1	DCA	2.799e-50
S1_1	Glycine	6.543e-135
S1_1	Paraformaldehyde	6.133e-1
S1_1	Nitrogen	9.950e-1
S1_1	Triethylamine	4.914e-18
S1_1	Aminoacetonitrile	3.028e-36
S1_1	Phosphoric_Acid	8.499e-107
S1_1	Ammonium_Chloride	4.145e-96

Table S8: Conversion Factor for Reaction Unit in L2 Structure.

j_{L2}	c	$\chi_{j^{L2},c}$
R1_1	Water	1.884
R1_1	Ammonia	-0.628
R1_1	Methane	-0.628
R1_1	HCN	0.628
R1_1	Oxygen	-0.942
R2_1	Water	0.946
R2_1	Ammonia	-0.571
R2_1	Formaldehyde	-0.946
R2_1	HCN	-0.946
R2_1	IDAN	0.375
R2_1	Aminoacetonitrile	0.196
R3_1	Water	-1.625
R3_1	Ammonia	1.625
R3_1	NaOH	-1.625
R3_1	IDAN	-0.813
R3_1	DSIDA	0.813
R1_4	Phosphorus	-0.157
R1_4	Chlorine	-0.940
R1_4	PCL3	0.627
R1_5	Water	-1.758
R1_5	Formaldehyde	-0.879
R1_5	HCl	0.879
R1_5	NaCl	1.758
R1_5	PCL3	-0.879
R1_5	DSIDA	-0.879
R1_5	PMIDA	0.879
R2_5	Formaldehyde	0.960
R2_5	PMG	0.960
R2_5	PMIDA	-0.960

R2_5	Oxygen	-0.480
R2_5	CO2	0.960
R1_2	Ammonia	-0.608
R1_2	EO	-0.986
R1_2	MEA	0.328
R1_2	DEA	0.182
R1_2	TEA	0.098
R2_2	NaOH	-1.890
R2_2	DEA	-0.945
R2_2	DSIDA	0.945
R2_2	Hydrogen	3.780
R1_3	HCl	1.900
R1_3	PCL3	-0.950
R1_3	Ethanol	-2.850
R1_3	Ethyl_Chloride	0.950
R1_3	DEP	0.950
R2_3	HCl	0.998
R2_3	Chlorine	-0.998
R2_3	Acetic_Acid	-0.971
R2_3	MCA	0.945
R2_3	DCA	0.026
R3_3	Ammonia	-1.840
R3_3	MCA	-0.920
R3_3	Glycine	0.920
R3_3	Ammonium_Chloride	0.920
R4_3	Water	-0.781
R4_3	PMG	0.781
R4_3	Ethanol	1.563
R4_3	DEP	-0.781
R4_3	Glycine	-0.781
R4_3	Paraformaldehyde	-0.781

Table S9: Feed Cost Factor.

								$FCF_{i^{L1},t}$								
t	StWater	StNH3	StForma	StNaOH	StHCl	StP	StCl2	StNG	StOxygen	StEO	StEtOH	StMeOH	StAcOH	StAc2O	StPFA	StEt3N
1	1.865e+0	1.122e+3	4.500e+2	3.462e+2	1.622e+2	5.020e+3	5.347e+2	7.059e+2	5.745e+1	1.440e+3	8.318e+2	6.600e+2	8.700e+2	1.100e+3	1.202e+3	2.960e+3
2	1.798e+0	1.122e+3	4.500e+2	3.462e+2	1.622e+2	5.020e+3	5.347e+2	7.059e+2	5.745e+1	1.440e+3	8.318e+2	6.600e+2	8.700e+2	1.100e+3	1.202e+3	2.960e+3
3	1.744e+0	1.122e+3	4.500e+2	3.462e+2	1.622e+2	5.020e+3	5.347e+2	7.059e+2	5.745e+1	1.440e+3	8.318e+2	6.600e+2	8.700e+2	1.100e+3	1.202e+3	2.960e+3
4	1.673e+0	1.122e+3	4.500e+2	3.462e+2	1.622e+2	5.020e+3	5.347e+2	7.059e+2	5.745e+1	1.440e+3	8.318e+2	6.600e+2	8.700e+2	1.100e+3	1.202e+3	2.960e+3
5	1.693e+0	1.122e+3	4.500e+2	3.462e+2	1.622e+2	5.020e+3	5.347e+2	7.059e+2	5.745e+1	1.440e+3	8.318e+2	6.600e+2	8.700e+2	1.100e+3	1.202e+3	2.960e+3
6	1.666e+0	1.189e+3	4.500e+2	3.559e+2	1.728e+2	5.090e+3	5.498e+2	8.387e+2	5.785e+1	1.500e+3	7.312e+2	6.400e+2	8.400e+2	1.100e+3	1.221e+3	3.007e+3
7	1.648e+0	1.189e+3	4.500e+2	3.559e+2	1.728e+2	5.090e+3	5.498e+2	8.387e+2	5.785e+1	1.500e+3	7.312e+2	6.400e+2	8.400e+2	1.100e+3	1.221e+3	3.007e+3
8	1.617e+0	1.189e+3	4.500e+2	3.559e+2	1.728e+2	5.090e+3	5.498e+2	8.387e+2	5.785e+1	1.500e+3	7.312e+2	6.400e+2	8.400e+2	1.100e+3	1.221e+3	3.007e+3
9	1.616e+0	1.189e+3	4.500e+2	3.559e+2	1.728e+2	5.090e+3	5.498e+2	8.387e+2	5.785e+1	1.500e+3	7.312e+2	6.400e+2	8.400e+2	1.100e+3	1.221e+3	3.007e+3
10	1.567e+0	1.262e+3	4.800e+2	3.625e+2	1.766e+2	5.060e+3	5.600e+2	7.545e+2	6.778e+1	1.510e+3	7.999e+2	6.400e+2	7.800e+2	1.110e+3	1.268e+3	3.124e+3
11	1.616e+0	1.262e+3	4.800e+2	3.625e+2	1.766e+2	5.060e+3	5.600e+2	7.545e+2	6.778e+1	1.510e+3	7.999e+2	6.400e+2	7.800e+2	1.110e+3	1.268e+3	3.124e+3
12	1.641e+0	1.262e+3	4.800e+2	3.625e+2	1.766e+2	5.060e+3	5.600e+2	7.545e+2	6.778e+1	1.510e+3	7.999e+2	6.400e+2	7.800e+2	1.110e+3	1.268e+3	3.124e+3
13	1.663e+0	1.262e+3	4.800e+2	3.625e+2	1.766e+2	5.060e+3	5.600e+2	7.545e+2	6.778e+1	1.510e+3	7.999e+2	6.400e+2	7.800e+2	1.110e+3	1.268e+3	3.124e+3
14	1.649e+0	1.370e+3	5.400e+2	3.793e+2	1.846e+2	4.980e+3	5.859e+2	8.518e+2	6.781e+1	1.560e+3	8.239e+2	6.800e+2	7.200e+2	1.120e+3	1.282e+3	3.157e+3
15	1.616e+0	1.370e+3	5.400e+2	3.793e+2	1.846e+2	4.980e+3	5.859e+2	8.518e+2	6.781e+1	1.560e+3	8.239e+2	6.800e+2	7.200e+2	1.120e+3	1.282e+3	3.157e+3
16	1.605e+0	1.370e+3	5.400e+2	3.793e+2	1.846e+2	4.980e+3	5.859e+2	8.518e+2	6.781e+1	1.560e+3	8.239e+2	6.800e+2	7.200e+2	1.120e+3	1.282e+3	3.157e+3
17	1.538e+0	1.370e+3	5.400e+2	3.793e+2	1.846e+2	4.980e+3	5.859e+2	8.518e+2	6.781e+1	1.560e+3	8.239e+2	6.800e+2	7.200e+2	1.120e+3	1.282e+3	3.157e+3
18	1.509e+0	1.358e+3	5.400e+2	4.046e+2	2.193e+2	4.870e+3	6.250e+2	1.017e+3	6.787e+1	1.530e+3	9.076e+2	6.500e+2	6.900e+2	1.130e+3	1.337e+3	3.293e+3
19	1.444e+0	1.358e+3	5.400e+2	4.046e+2	2.193e+2	4.870e+3	6.250e+2	1.017e+3	6.787e+1	1.530e+3	9.076e+2	6.500e+2	6.900e+2	1.130e+3	1.337e+3	3.293e+3
20	1.473e+0	1.358e+3	5.400e+2	4.046e+2	2.193e+2	4.870e+3	6.250e+2	1.017e+3	6.787e+1	1.530e+3	9.076e+2	6.500e+2	6.900e+2	1.130e+3	1.337e+3	3.293e+3
21	1.494e+0	1.358e+3	5.400e+2	4.046e+2	2.193e+2	4.870e+3	6.250e+2	1.017e+3	6.787e+1	1.530e+3	9.076e+2	6.500e+2	6.900e+2	1.130e+3	1.337e+3	3.293e+3
22	1.519e+0	1.358e+3	5.400e+2	4.046e+2	2.193e+2	4.870e+3	6.250e+2	1.017e+3	6.787e+1	1.530e+3	9.076e+2	6.500e+2	6.900e+2	1.130e+3	1.337e+3	3.293e+3
23	1.503e+0	1.279e+3	5.400e+2	4.138e+2	2.303e+2	4.810e+3	6.392e+2	1.184e+3	6.792e+1	1.540e+3	9.401e+2	6.300e+2	6.800e+2	1.160e+3	1.355e+3	3.339e+3
24	1.358e+0	1.279e+3	5.400e+2	4.138e+2	2.303e+2	4.810e+3	6.392e+2	1.184e+3	6.792e+1	1.540e+3	9.401e+2	6.300e+2	6.800e+2	1.160e+3	1.355e+3	3.339e+3
25	1.354e+0	1.279e+3	5.400e+2	4.138e+2	2.303e+2	4.810e+3	6.392e+2	1.184e+3	6.792e+1	1.540e+3	9.401e+2	6.300e+2	6.800e+2	1.160e+3	1.355e+3	3.339e+3
26	1.391e+0	1.279e+3	5.400e+2	4.138e+2	2.303e+2	4.810e+3	6.392e+2	1.184e+3	6.792e+1	1.540e+3	9.401e+2	6.300e+2	6.800e+2	1.160e+3	1.355e+3	3.339e+3
27	1.394e+0	1.130e+3	5.300e+2	4.534e+2	2.221e+2	4.980e+3	7.003e+2	1.052e+3	8.004e+1	1.460e+3	8.888e+2	6.200e+2	6.700e+2	1.190e+3	1.356e+3	3.341e+3
28	1.378e+0	1.130e+3	5.300e+2	4.534e+2	2.221e+2	4.980e+3	7.003e+2	1.052e+3	8.004e+1	1.460e+3	8.888e+2	6.200e+2	6.700e+2	1.190e+3	1.356e+3	3.341e+3
29	1.427e+0	1.130e+3	5.300e+2	4.534e+2	2.221e+2	4.980e+3	7.003e+2	1.052e+3	8.004e+1	1.460e+3	8.888e+2	6.200e+2	6.700e+2	1.190e+3	1.356e+3	3.341e+3
30	1.481e+0	1.130e+3	5.300e+2	4.534e+2	2.221e+2	4.980e+3	7.003e+2	1.052e+3	8.004e+1	1.460e+3	8.888e+2	6.200e+2	6.700e+2	1.190e+3	1.356e+3	3.341e+3
31	1.529e+0	1.130e+3	5.300e+2	4.534e+2	2.221e+2	4.980e+3	7.003e+2	1.052e+3	8.004e+1	1.460e+3	8.888e+2	6.200e+2	6.700e+2	1.190e+3	1.356e+3	3.341e+3
32	1.554e+0	1.110e+3	5.300e+2	4.609e+2	2.028e+2	5.110e+3	7.120e+2	1.274e+3	7.976e+1	1.390e+3	8.539e+2	6.200e+2	6.800e+2	1.190e+3	1.279e+3	3.151e+3
33	1.570e+0	1.110e+3	5.300e+2	4.609e+2	2.028e+2	5.110e+3	7.120e+2	1.274e+3	7.976e+1	1.390e+3	8.539e+2	6.200e+2	6.800e+2	1.190e+3	1.279e+3	3.151e+3
34	1.502e+0	1.110e+3	5.300e+2	4.609e+2	2.028e+2	5.110e+3	7.120e+2	1.274e+3	7.976e+1	1.390e+3	8.539e+2	6.200e+2	6.800e+2	1.190e+3	1.279e+3	3.151e+3
35	1.439e+0	1.110e+3	5.300e+2	4.609e+2	2.028e+2	5.110e+3	7.120e+2	1.274e+3	7.976e+1	1.390e+3	8.539e+2	6.200e+2	6.800e+2	1.190e+3	1.279e+3	3.151e+3
36	1.438e+0	1.096e+3	4.700e+2	4.710e+2	2.057e+2	5.490e+3	7.276e+2	1.379e+3	7.997e+1	1.330e+3	8.512e+2	6.100e+2	6.700e+2	1.160e+3	1.245e+3	3.068e+3
37	1.435e+0	1.096e+3	4.700e+2	4.710e+2	2.057e+2	5.490e+3	7.276e+2	1.379e+3	7.997e+1	1.330e+3	8.512e+2	6.100e+2	6.700e+2	1.160e+3	1.245e+3	3.068e+3
38	1.366e+0	1.096e+3	4.700e+2	4.710e+2	2.057e+2	5.490e+3	7.276e+2	1.379e+3	7.997e+1	1.330e+3	8.512e+2	6.100e+2	6.700e+2	1.160e+3	1.245e+3	3.068e+3
39	1.289e+0	1.096e+3	4.700e+2	4.710e+2	2.057e+2	5.490e+3	7.276e+2	1.379e+3	7.997e+1	1.330e+3	8.512e+2	6.100e+2	6.700e+2	1.160e+3	1.245e+3	3.068e+3
40	1.337e+0	1.157e+3	4.200e+2	4.935e+2	2.051e+2	5.700e+3	7.624e+2	1.181e+3	7.991e+1	1.250e+3	8.305e+2	6.000e+2	6.700e+2	1.150e+3	1.221e+3	3.007e+3
41	1.288e+0	1.157e+3	4.200e+2	4.935e+2	2.051e+2	5.700e+3	7.624e+2	1.181e+3	7.991e+1	1.250e+3	8.305e+2	6.000e+2	6.700e+2	1.150e+3	1.221e+3	3.007e+3
42	1.331e+0	1.157e+3	4.200e+2	4.935e+2	2.051e+2	5.700e+3	7.624e+2	1.181e+3	7.991e+1	1.250e+3	8.305e+2	6.000e+2	6.700e+2	1.150e+3	1.221e+3	3.007e+3
43	1.399e+0	1.157e+3	4.200e+2	4.935e+2	2.051e+2	5.700e+3	7.624e+2	1.181e+3	7.991e+1	1.250e+3	8.305e+2	6.000e+2	6.700e+2	1.150e+3	1.221e+3	3.007e+3
44	1.401e+0	1.157e+3	4.200e+2	4.935e+2	2.051e+2	5.700e+3	7.624e+2	1.181e+3	7.991e+1	1.250e+3	8.305e+2	6.000e+2	6.700e+2	1.150e+3	1.221e+3	3.007e+3
45	1.443e+0	1.191e+3	3.900e+2	4.944e+2	1.633e+2	5.910e+3	7.636e+2	9.632e+2	7.993e+1	1.200e+3	8.687e+2	6.000e+2	6.400e+2	1.140e+3	1.206e+3	2.970e+3
46	1.501e+0	1.191e+3	3.900e+2	4.944e+2	1.633e+2	5.910e+3	7.636e+2	9.632e+2	7.993e+1	1.200e+3	8.687e+2	6.000e+2	6.400e+2	1.140e+3	1.206e+3	2.970e+3
47	1.534e+0	1.191e+3	3.900e+2	4.944e+2	1.633e+2	5.910e+3	7.636e+2	9.632e+2	7.993e+1	1.200e+3	8.687e+2	6.000e+2	6.400e+2	1.140e+3	1.206e+3	2.970e+3
48	1.527e+0	1.191e+3	3.900e+2	4.944e+2	1.633e+2	5.910e+3	7.636e+2	9.632e+2	7.993e+1	1.200e+3	8.687e+2	6.000e+2	6.400e+2	1.140e+3	1.206e+3	2.970e+3
49	1.521e+0	1.150e+3	4.600e+2	4.920e+2	1.612e+2	5.620e+3	7.600e+2	1.045e+3	8.000e+1	1.180e+3	8.100e+2	5.800e+2	6.300e+2	1.140e+3	1.187e+3	2.923e+3
50	1.522e+0	1.150e+3	4.600e+2	4.920e+2	1.612e+2	5.620e+3	7.600e+2	1.045e+3	8.000e+1	1.180e+3	8.100e+2	5.800e+2	6.300e+2	1.140e+3	1.187e+3	2.923e+3
51	1.477e+0	1.150e+3	4.600e+2	4.920e+2	1.612e+2	5.620e+3	7.600e+2	1.045e+3	8.000e+1	1.180e+3	8.100e+2	5.800e+2	6.300e+2	1.140e+3	1.187e+3	2.923e+3
52	1.481e+0	1.150e+3	4.600e+2	4.920e+2	1.612e+2	5.620e+3	7.600e+2	1.045e+3	8.000e+1	1.180e+3	8.100e+2	5.800e+2	6.300e+2	1.140e+3	1.187e+3	2.923e+3

Table S10: L1 Feed Stream Inlet Compositions.

i^{L1}	c	$z^{L1}_{i^{L1},c}$
StNG	Methane	0.996
StNG	Nitrogen	0.004
StAir	Oxygen	0.210
StAir	Nitrogen	0.790
StNH3	Ammonia	1.000
StForma	Formaldehyde	0.275
StForma	Water	0.656
StForma	Methanol	0.070
StPA	Phosphoric_Acid	0.510
StPA	Water	0.490
StNaOH	NaOH	0.311
StNaOH	Water	0.689
StWater	Water	1.000
StOxygen	Oxygen	1.000
StP	Phosphorus	1.000
StCl2	Chlorine	1.000
StEO	EO	1.000
StAcOH	Acetic_Acid	1.000
StAc2O	Acetic_Anhydride	1.000
StEtOH	Ethanol	1.000
StHCl	Hcl	0.225
StHCl	Water	0.775
StPFA	Paraformaldehyde	0.951
StPFA	Water	0.049
StMeOH	Methanol	1.000
StEt3N	Triethylamine	1.000

Table S11: Superstructure L1 Block Parameters.

j^{L1}	τ	$P^{min}_{j^{L1},\tau}$	$P^{max}_{j^{L1},\tau}$	$A^{PC}_{j^{L1},\tau}$	$B^{PC}_{j^{L1},\tau}$
B_PCl3	P_HCN	0.268	5.351	1.126e+4	0.6
B_HCN	P_HCN	0.810	16.196	1.415e+5	0.6
B_IDAN	P_HCN	1.048	20.953	1.994e+5	0.6
B_DSIDA1	P_HCN	7.970	159.406	5.493e+4	0.6
B_PMIDA	P_HCN	29.576	591.518	6.400e+3	0.6
B_PMG1	P_HCN	0.168	3.360	2.757e+4	0.6
B_PCl3	P_DEA	0.268	5.351	1.126e+4	0.6
B_DEA	P_DEA	0.216	4.323	7.081e+4	0.6
B_DSIDA2	P_DEA	0.318	6.369	1.008e+4	0.6
B_PMIDA	P_DEA	29.446	588.923	1.119e+3	0.6
B_PMG1	P_DEA	0.168	3.360	6.848e+4	0.6
B_PCl3	P_Glycine	0.229	4.572	1.164e+4	0.6
B_DEPP	P_Glycine	0.179	3.576	2.314e+4	0.6
B_Glycine	P_Glycine	1.380	27.606	1.112e+4	0.6
B_PMG2	P_Glycine	0.168	3.360	1.594e+5	0.6

Table S12: Inverse Molar Densities.

$i^{L1_{feed}}$	$(\rho^W_{i^{L1}})^{-1}$
StNG	37.462
StAir	26674.736
StNH3	24.954
StForma	34.704
StPA	74.992
StNaOH	34.215
StWater	18.074
StOxygen	28.018
StP	13.237
StCl2	45.434
StEO	49.279
StAcOH	57.632
StAc2O	95.040
StEtOH	58.620
StHCl	46.701
StPFA	34.785
StMeOH	40.579
StEt3N	139.672
$(\rho^S)^{-1}$	99.453

Table S13: Reaction Chemical Ratios.

i^{L2}	c	$Ratio^1_{i^{L2},c}$	$Ratio^2_{i^{L2},c}$
St6_4	Phosphorus	3.958	1
St6_4	Chlorine	1.000	1
St4_3	PCL3	3.800	1
St4_3	Ethanol	1.000	1
St16_3	Chlorine	1.000	1
St16_3	Acetic_Anhydride	3.387	1
St16_3	Acetic_Acid	1.000	1
St29_3	Ammonia	0.333	1
St29_3	Formaldehyde	0.667	1
St29_3	MCA	1.000	1
St42_3	HCl	0.219	1
St42_3	Methanol	0.041	1
St42_3	DEP	1.254	1
St42_3	Glycine	1.000	1
St42_3	Paraformaldehyde	0.500	1
St42_3	Triethylamine	1.076	1
St10_2	Water	0.201	1
St10_2	Ammonia	0.359	1
St10_2	EO	1.000	1
St34_2	Water	0.073	1
St34_2	NaOH	0.468	1
St34_2	DEA	1.000	1
St5_4	Phosphorus	3.958	1
St5_4	Chlorine	1.000	1
St5_5	Formaldehyde	0.841	1
St5_5	HCl	2.234	1
St5_5	PCL3	0.846	1
St5_5	DSIDA	1.000	1
St9_5	Water	0.006	1
St9_5	PMIDA	1.000	1
St16_5	PMIDA	1.000	1
St16_5	Oxygen	0.333	1
St7_1	Ammonia	1.693	1
St7_1	Methane	1.000	1
St7_1	Oxygen	0.896	1
St25_1	Ammonia	0.244	1
St25_1	Formaldehyde	1.000	1
St25_1	HCN	1.000	1
St25_1	Phosphoric_Acid	32.951	1
St42_1	Water	0.003	1
St42_1	NaOH	0.500	1
St42_1	IDAN	1.000	1

Table S14: L2 Stream Feed Compositions.

i^{L2}	c	$z_{i^{L2},c}$
St1_4	Phosphorus	1.000
St3_4	Chlorine	1.000
St1_3	Ethanol	1.000
St12_3	Acetic_Acid	1.000
St13_3	Acetic_Anhydride	1.000
St14_3	Chlorine	1.000
St25_3	Ammonia	1.000
St27_3	Water	0.656
St27_3	Formaldehyde	0.275
St27_3	Methanol	0.070
St36_3	Triethylamine	1.000
St34_3	Water	0.049
St34_3	Paraformaldehyde	0.951
St35_3	Methanol	1.000
St39_3	Water	0.775
St39_3	HCl	0.225
St1_2	EO	1.000
St4_2	Ammonia	1.000
St7_2	Water	1.000
St29_2	Water	1.000
St30_2	Water	0.689
St30_2	NaOH	0.311
St2_5	Water	0.656
St2_5	Formaldehyde	0.275
St2_5	Methanol	0.070
St3_5	Water	0.775
St3_5	HCl	0.225
St8_5	Water	1.000
St11_5	Oxygen	1.000
St4_1	Methane	0.996
St4_1	Nitrogen	0.004
St1_1	Oxygen	0.210
St1_1	Nitrogen	0.790
St2_1	Ammonia	1.000
St10_1	Water	1.000
St18_1	Water	0.490
St18_1	Phosphoric_Acid	0.510
St19_1	Ammonia	1.000
St21_1	Water	0.656
St21_1	Formaldehyde	0.275
St21_1	Methanol	0.070
St40_1	Water	1.000
St39_1	Water	0.689
St39_1	NaOH	0.311

Table S15: Molecular Weight of Chemicals.

c	MW_c
Water	18.015
Ammonia	17.031
Formaldehyde	30.026
NaOH	39.9967693
HCl	36.458
NaCl	58.4397693
Phosphorus	123.895048
Chlorine	70.9
PCL3	137.323762
PMG	169.072762
Methanol	32.042
Ethanol	46.069
Methane	16.043
HCN	27.026
EO	44.053
MEA	61.084
DEA	105.137
TEA	149.19
IDAN	95.105
DSIDA	177.0665386
PMIDA	227.108762
Hydrogen	2.016
Oxygen	31.998
CO2	44.009
Ethyl_Chloride	64.512
DEP	138.102762
Acetic_Anhydride	102.089
Acetic_Acid	60.052
MCA	94.494
DCA	128.936
Glycine	75.067
Paraformaldehyde	30.026
Nitrogen	28.014
Triethylamine	101.193
Aminoacetonitrile	56.068
Phosphoric_Acid	97.993762
Ammonium_Chloride	53.489

Table S16: Demand Flowrate $Demand_t$.

t	Scenario 1	Scenario 2
1	0.628	0.295
2	0.606	0.278
3	0.706	0.324
4	0.786	0.359
5	1.300	0.600
6	0.739	0.333
7	0.508	0.232
8	0.415	0.190
9	0.918	0.425
10	0.484	0.225
11	0.556	0.249
12	0.555	0.255
13	0.787	0.364
14	0.675	0.312
15	0.607	0.278
16	0.566	0.260
17	0.783	0.368
18	1.252	0.592
19	0.867	0.399
20	0.842	0.393
21	0.922	0.432
22	1.306	0.609
23	0.846	0.396
24	1.110	0.517
25	1.101	0.516
26	1.519	0.726
27	1.122	0.522
28	1.331	0.625
29	1.581	0.746
30	1.336	0.618
31	1.378	0.656
32	0.730	0.347
33	0.538	0.252
34	0.489	0.230
35	0.646	0.311
36	0.300	0.141
37	0.345	0.163
38	0.313	0.147
39	0.663	0.315
40	0.371	0.169
41	0.343	0.161
42	0.425	0.199
43	0.398	0.189
44	0.663	0.316
45	0.292	0.138
46	0.562	0.260
47	0.403	0.189
48	0.505	0.238

49	0.320	0.155
50	0.253	0.119
51	0.486	0.228
52	0.104	0.048

References

[1] *Aspen HYSYS;* AspenTech, 2024.

[2] Stephenson, R. M.; Malanowski, S. *Handbook of the Thermodynamics of Organic Compounds*; Springer Netherlands, 1987.

[3] Chang, F. C.; Simcik, M. F.; Capel, P. D. Occurrence and fate of the herbicide glyphosate and its degradate aminomethylphosphonic acid in the atmosphere. *Environmental Toxicology and Chemistry* **2011**, *30*, 548–555.

[4] ChemBK. *ChemBK: Chemical Information and Data Search.* https://www.chembk.com/en.

[5] EnvModels. *EnvModels – Environmental Modeling Software.* https://www.envmodels.com.

[6] INCHEM. *International Peer Reviewed Chemical Safety Information.* https://www.inchem.org.

[7] CHEMSRC. *CHEMSRC.* https://www.chemsrc.com/en/.

[8] ECHEMI. *ECHEMI.* https://www.echemi.com/.

[9] NIH. *PubChem.* https://pubchem.ncbi.nlm.nih.gov/.

[10] Linstrom, P., Mallard, W., Eds. *NIST Chemistry WebBook, NIST Standard Reference Database Number 69*; National Institute of Standards and Technology, Gaithersburg MD, 20899.

[11] Chemeo. *Chemeo.* https://www.chemeo.com/.

[12] Toolbox., T. E. The Engineering Toolbox. https://www.engineeringtoolbox.com/.

[13] WebElements. *Phosphorus: Thermochemistry.* https://www.webelements.com/phosphorus/thermochemistry.html.

[14] Perry, R.; Green, D.; Maloney, J. *Perry's Chemical Engineers' Handbook*; International student edition v. 7, pt. 1997; McGraw-Hill, 1997.

[15] Grigoras, S. A structural approach to calculate physical properties of pure organic substances: The critical temperature, critical volume and related properties. *Journal of Computational Chemistry* **1990**, *11*, 493–510.

[16] Hurst, J. E.; Harrison, B. K. Estimation of liquid and solid heat capacities using a modified kopp's rule. *Chemical Engineering Communications* **1992**, *112*, 21–30.

[17] Mersmann, A.; Kind, M. Correlation for the Prediction of Critical Molar Volume. *Industrial and Engineering Chemistry Research* **2017**, *56*, 13970–13971.

[18] Gail, E.; Gos, S.; Kulzer, R.; Lorösch, J.; Rubo, A.; Sauer, M.; Kellens, R.; Reddy, J.; Steier, N.; Hasenpusch, W. *Ullmann's Encyclopedia of Industrial Chemistry*; Wiley, 2011; Chapter Cyano Compounds, Inorganic.

[19] Royal Society of Chemistry. *Periodic Table.* https://periodic-table.rsc.org/.

[20] Seider, W. D.; Lewin, D. R.; Seader, J.; Widagdo, S.; Gani, R.; Ng, K. M. *Product and process design principles: synthesis, analysis and evaluation*; John Wiley & Sons, 2016.

[21] Gavin Towler, R. S. *Chemical Engineering Design: Principles, Practice And Economics of Plant And Process Design*; Elsevier, 2022.

[22] Davis, R. A. Gilliland's Correlation: A Case Study in Regression Analysis. *Chemical Engineering Education* **2020**, *54*.

[23] Martin, J. J.; Edwards, J. B. Correlation of latent heats of vaporization. *AIChE Journal* **1965**, *11*, 331–333.

[24] Anonymous Economic Indicators. *Chemical Engineering* **2024**, *131*, 60, Copyright - Copyright Access Intelligence LLC Apr 2024 Last updated - 2024-08-25.

[25] Williams Jr., R. "Six-Tenths Factor" Aids in Approximating Costs. *Chemical Engineering* **1947**, *54*, 124–125.
